# Epithelial-mesenchymal transition (EMT) signature is inversely associated with T-cell infiltration in non-small cell lung cancer (NSCLC)

**DOI:** 10.1038/s41598-018-21061-1

**Published:** 2018-02-13

**Authors:** Young Kwang Chae, Sangmin Chang, Taeyeong Ko, Jonathan Anker, Sarita Agte, Wade Iams, Wooyoung M. Choi, Kyoungmin Lee, Marcelo Cruz

**Affiliations:** 1 Northwestern University Feinberg School of Medicine, Department of Medicine, Chicago, IL USA; 2 Department of Internal Medicine, Asan Medical Center, University of Ulsan College of Medicine, Seoul, Republic of Korea

## Abstract

Epithelial-mesenchymal transition (EMT) is able to drive metastasis during progression of multiple cancer types, including non-small cell lung cancer (NSCLC). As resistance to immunotherapy has been associated with EMT and immune exclusion in melanoma, it is important to understand alterations to T-cell infiltration and the tumor microenvironment during EMT in lung adenocarcinoma and squamous cell carcinoma. We conducted an integrated analysis of the immune landscape in NSCLCs through EMT scores derived from a previously established 16 gene signature of canonical EMT markers. EMT was associated with exclusion of immune cells critical in the immune response to cancer, with significantly lower infiltration of CD4 T-cells in lung adenocarcinoma and CD4/CD8 T-cells in squamous cell carcinoma. EMT was also associated with increased expression of multiple immunosuppressive cytokines, including IL-10 and TGF-β. Furthermore, overexpression of targetable immune checkpoints, such as CTLA-4 and TIM-3 were associated with EMT in both NSCLCs. An association may exist between immune exclusion and EMT in NSCLC. Further investigation is merited as its mechanism is not completely understood and a better understanding of this association could lead to the development of biomarkers that could accurately predict response to immunotherapy.

## Introduction

A plethora of studies have shown that the innate and adaptive immune systems play a crucial role in the anticancer response as they recognize and destroy cancer cells by a process known as cancer immunosurveillance^1,2^. The major constituents of this defense system are tumor antigen-specific cytotoxic T-lymphocytes (CTLs), whose anti-tumor functions are amplified by immune checkpoint blockade antibodies such as CTLA-4 inhibitors and PD-1/PD-L1 inhibitors^3–6^. Though such treatment modalities have shown success in select cancer types including melanoma and non-small cell lung cancer (NSCLC), the majority of cancer types are unresponsive to these treatments. Even so, only a subset of patients can be treated due to the low baseline level of CD8 T-cell infiltration within the tumor microenvironment (TME) necessary to achieve therapeutic benefit^5,7,8^. The lack of such effector cells in the TME is known as immune exclusion^9^, which has been known to be mediated by the β-catenin pathway in melanoma, leading to resistance to immunotherapy^7,10–12^. Moreover, improved outcome has been shown to be highly correlated with the presence of lymphocytic infiltrates within the tumor^8^.

In melanoma, the subset of tumors inherently resistant to immunotherapy were shown to display innate PD-1 resistance (IPRES), a transcriptional signature of overexpressed genes involved in the regulation of epithelial-mesenchymal transition (EMT), immunosuppression, angiogenesis, and monocyte and macrophage chemotaxis^13^. In particular, treatment-resistant tumors displayed decreased expression of *CDH1*, an epithelial marker gene, and increased expression of the T-cell suppressive cytokine, *IL10*. This was consistent with previous findings of immunotherapy-resistant melanoma patients having increased gene expression patterns associated with EMT, along with lower T-cell infiltration^14,15^.

In contrast to the mechanisms of immunotherapy resistance in melanoma, such pathways in NSCLC remain largely unknown^5,6^. However, as with melanoma, EMT in NSCLC has been associated with disease progression and poor prognosis^16,17^, as well as immune evasion in lung adenocarcinoma (ADC)^10,12,18^. EMT was furthermore found to have a molecular link with intratumoral CD8 T-cell suppression through ZEB1, which activates EMT and represses microRNA-200, a suppressor of EMT and PD-L1^18,19^. Therefore, the relationship between immune exclusion of tumor-infiltrating T-cells and EMT is of great interest in NSCLC.

While this relationship has been explored in lung ADC, this has not been the case with lung squamous cell carcinoma (SqCC). As we expected similar results between lung ADC and SqCC, we conducted an integrated analysis of the relationship between the expression of signature EMT gene markers and their associated tumor immunophenotype, in both lung ADC and SqCC. Given that infiltration of T-cells in the TME impacts response to immune checkpoint inhibitors, understanding the association between immune exclusion, especially that of CD8 T-cells, and EMT would be of value in the development of biomarkers capable of accurately predicting response to immunotherapeutic modalities.

## Methods

### Gene expression analysis to determine EMT scores, tumor immune landscape, CD8 T-cell score, and cytokine and immune checkpoint gene analysis

**Figure 1:**
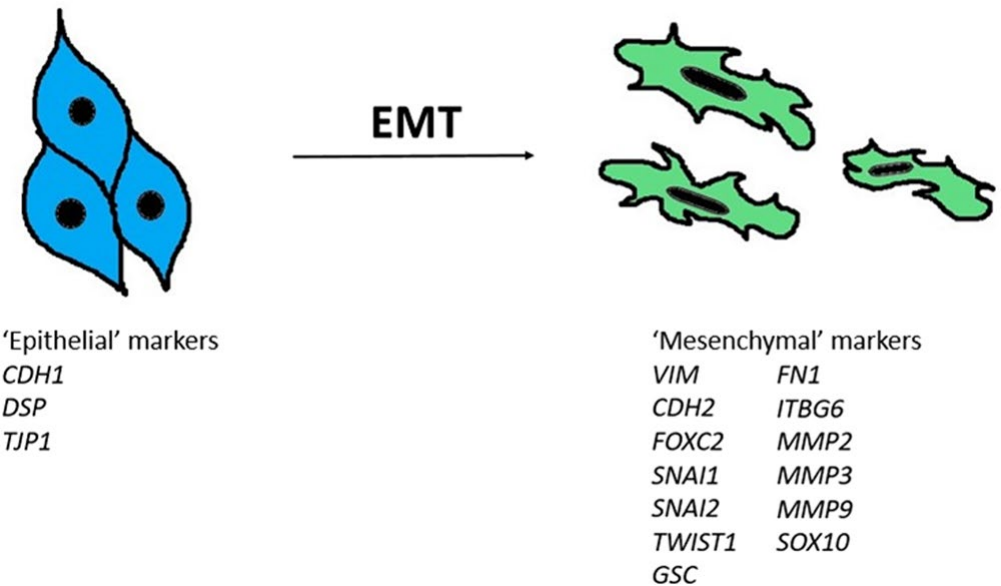
16 canonical gene markers of EMT. The 16 canonical genes consist of 3 ‘epithelial’ and 13 ‘mesenchymal’ genes.

Gene expression RNA-sequencing z-scores were obtained from The Cancer Genome Atlas (TCGA) database using cBioPortal^20^ for a previously identified 16 canonical gene markers of EMT (Fig. 1)^21^ for lung ADC (515 samples) and lung SqCC (501 samples). One difference between the 16 gene signature of EMT markers used in this study from the previous study was the replacement of *OCLN*, an ‘epithelial’ marker for which data was not available, with *TJP1*, a marker of attenuation in EMT, and, hence, of ‘epithelial’ nature shown in previous studies^22,23^.

The tumor immune landscape was analyzed from RNA-seq z-scores of 812 ‘immune metagene’ signatures established as previously described, which were used to predict immune infiltration of 31 distinct immune cells for each tumor sample. Expression z-scores were used as input in the Gene Set Enrichment Analysis (GSEA) from the Broad Institute, and any immune cell type with a false discovery rate (q-value) ≤ 10% was considered to be positively infiltrating^24^.

EMT scores were generated by subtracting the average RNA-seq z-scores of 3 ‘epithelial’ marker genes from the average RNA-seq z-scores of 13 ‘mesenchymal’ marker genes for each sample. Patient samples were grouped as either EMT-high (highest 1/3 of EMT scores) or EMT-low (lowest 1/3 of EMT scores)^18^. The EMT-high group was defined as ‘mesenchymal’ lung ADC or SqCC tumors while the EMT-low group was defined as ‘epithelial’ ADC or SqCC. The infiltration of 31 distinct immune cells in ‘mesenchymal’ lung ADC (*n* = 172) was compared to that of ‘epithelial’ lung ADC (*n* = 171), while the same was done for mesenchymal’ (*n* = 167) and ‘epithelial’ (*n* = 167) lung SqCC.

The CD8 T-cell score was generated from the mean gene expression z-score of four CD8 T-cell signature markers (*CD8A, CD8B, IGNF*, and *PRF1*) as previously established^25^. CD8 T-cell scores were stratified as high (highest 1/3), intermediate (middle 1/3), and low (lowest 1/3).

The distribution of RNA-seq z-scores of 16 immunosuppressive and inflammatory cytokine genes (*IFNα*, *IFNβ*, *IFNγ*, *TNFα*, *TGFβ*, *IL1A*, *IL1B*, *IL2*, *IL3*, *IL-4*, *IL5*, *IL6*, *CXCL8*, *IL10*, *IL12A*, *IL12B*) as well as six immune checkpoint markers (*CD274* (*PD-L1*), *CTLA-4*, *HAVCR2* (*TIM3*), *ICOS*, *TNFRSF4 (OX40/CD134)*, *TNFRSF9* (*4-1BB/CD137))* and *FASLG (FasL/CD95L)* were compared between ‘mesenchymal’ and ‘epithelial’ groups. Expression of immune related cytokines and immune checkpoint genes were also analyzed utilizing univariate simple linear-regression analysis.

### Individual gene analysis

The immune landscapes of each of the 16 individual canonical gene markers of EMT were analyzed separately. Immune infiltrations of 31 distinct immune cells were compared for each high gene expression group (RNA-seq z-score ≥ highest 1/3) and low expression group (RNA-seq z-score ≤ lowest 1/3).

### Survival outcome

Overall survival (OS) data from TCGA was used to plot Kaplan-Meier survival curves. Survival of the ‘mesenchymal’ group (*n* = 167) was compared to the ‘epithelial’ group (*n* = 165) in lung ADC and lung SqCC (*n* = 162 each). Survival of infiltration of activated CD8 T-cells in the ‘mesenchymal’ and ‘epithelial’ groups were also analyzed. The OS of four groups were compared for lung ADC and SqCC, where the four groups were Mesenchymal/activated CD8 T-cell infiltration present (*n* = 39, 35 respectively), Mesenchymal/activated CD8 T-cell infiltration absent (128, 130 respectively), Epithelial/activated CD8 T-cell infiltration present (*n* = 48, 48 respectively), and Epithelial/activated CD8 T-cell infiltration absent (*n* = 120, 105 respectively).

### Statistical analysis

Statistical significance for infiltration of immune cells was computed using the chi-square test. Statistical significance for the gene expression of cytokine and immune checkpoint genes were computed using Welch’s t-test. Statistical significance for OS was computed by utilizing the log-rank test.

## Results

### EMT-high score was associated with lower CD4 T-cell infiltration in human lung ADC, lower CD4/CD8 T-cell infiltration in human lung SqCC, and higher infiltration of activated B cells and regulatory T-cells in human lung ADC and SqCC

**Figure 2:**
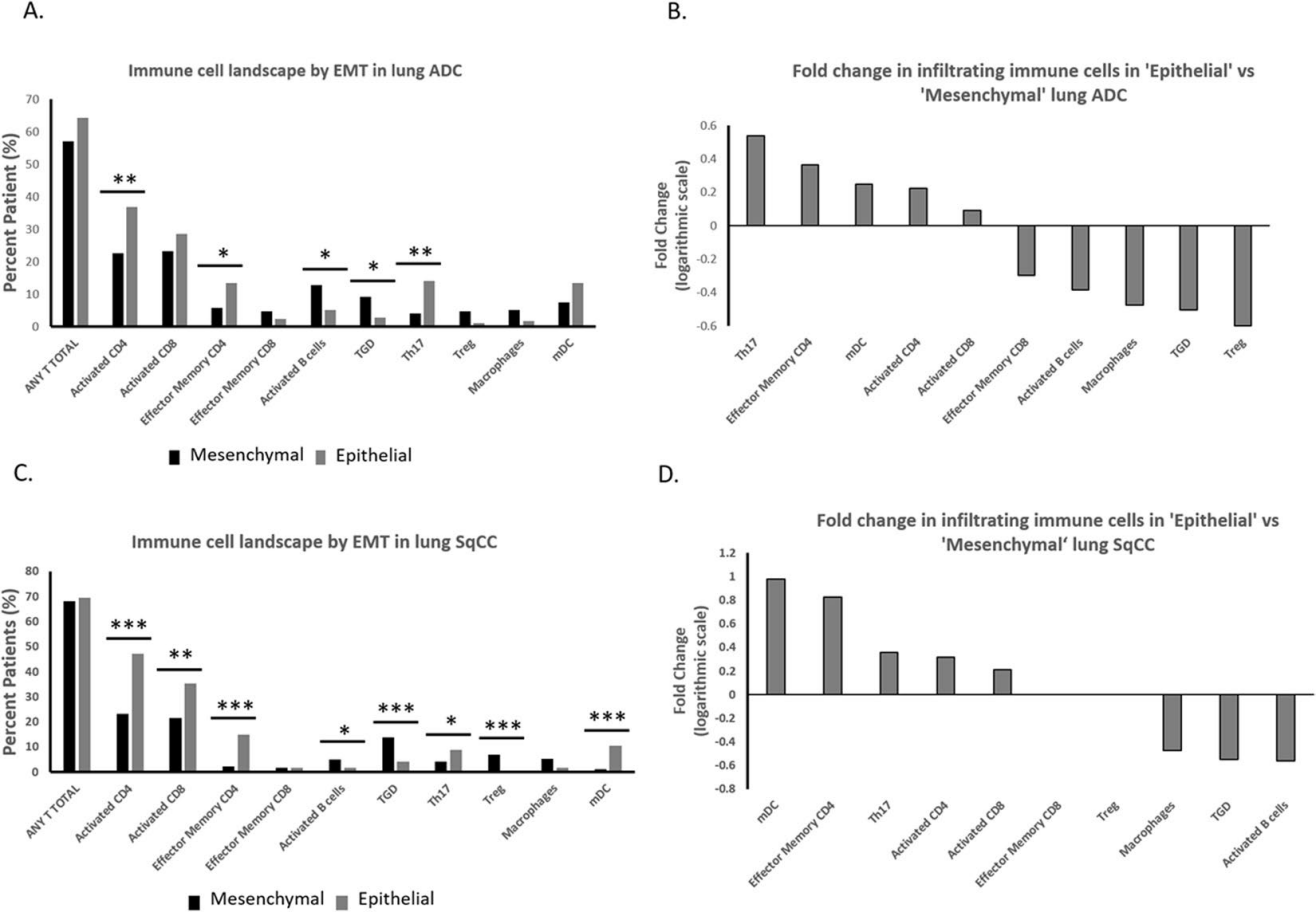
Immune cell infiltration landscape by EMT score status. (**A**) Immune infiltration of ‘mesenchymal’ lung ADC compared to ‘epithelial’ lung ADC. (**B**) Fold change of infiltration of immune cells compared between ‘epithelial’ and ‘mesenchymal’ lung ADC. (**C**) Immune infiltration of ‘mesenchymal’ lung SqCC compared to ‘epithelial’ lung SqCC. (**D**) Fold change of infiltration of immune cells compared between ‘epithelial’ and ‘mesenchymal’ lung SqCC. *p < *0.05*, **p < *0.01*, ***p < *0.001*.

As EMT has been implicated with intratumoral CD8 T-cell suppression, we sought to determine which immune cells were excluded from the TME in NSCLC. In ‘mesenchymal’ lung ADC, tumors displayed significantly decreased infiltration of activated CD4 T-cells, effector CD4 T-cells, and Th17 cells, with significantly higher infiltration of activated B-cells and γδ T-cells (TGD) (Fig. 2A). However, infiltration of activated CD8 T-cells and regulatory T-cells (Tregs) were not significantly different. The immune infiltration and overall immune landscape of 31 immune cells in lung ADC are presented in Supplementary Figure 1. In comparing the fold change of infiltration of immune cells based on EMT status, we observed an increased infiltration of cells with anti-tumor or immune stimulatory functions such as Th17, mature dendritic cells (mDC), and activated CD4 T-cells in ‘epithelial’ lung ADC. Similarly, cells with pro-tumor or immunosuppressive functions such as Tregs displayed decreased infiltration in ‘epithelial’ lung ADC (Fig. 2B). Therefore in ‘mesenchymal’ lung ADC, there was a pattern of immune exclusion of pro-tumor cells while anti-tumor cells showed increased infiltration.

We further conducted this analysis in lung SqCC. The immune landscape of lung SqCC was similar to that of lung ADC but showed more pronounced immune exclusion with significantly lower infiltration of activated CD4, activated CD8, and effector memory CD4 T-cells in ‘mesenchymal’ samples. Infiltration of Th17 and mDC were also significantly decreased in ‘mesenchymal’ lung SqCC tumors (Fig. 2C). On the contrary, activated B cells, TGD, and Tregs showed significantly increased infiltration in ‘mesenchymal’ lung SqCC tumors. The immune infiltration and overall immune landscape of 31 immune cells in lung SqCC are presented in Supplementary Figure 2. As in lung ADC, lung SqCC displayed increased infiltration by immune stimulatory cell types, such as mDC, Th17, and activated CD4 and 8 T-cells in ‘epithelial tumors. Cells with immunosuppressive functions such as Treg and TGD showed decreased infiltration in ‘epithelial’ lung SqCC (Fig. 2D). Overall, lung ADC and lung SqCC showed a similar pattern infiltrating cell types.

In terms of CD8 T-cell signature score, there was no discernable pattern of increased or decreased pattern of either mesenchymal or epithelial gene expression (RNA-seq z-score, Supplementary Figure 3).

### ‘Mesenchymal’ lung ADC and SqCC are associated with an inflammatory TME, with a general trend towards higher expression of immunosuppressive and inflammatory cytokines

Expression of 16 immunosuppressive and inflammatory cytokine genes were compared between ‘mesenchymal’ and ‘epithelial’ groups of lung ADC and SqCC tumors. Of particular interest were immunosuppressive cytokine markers *TGFB1 (TGF- β)* and *IL-10*, both of which showed significantly increased z-score distribution in ‘mesenchymal’ groups of lung ADC and SqCC (p 0.001 each). Similarly, linear-regression of *TGFB1 (TGF- β)* and *IL-10* z-scores both showed a weak positive correlation with EMT score (p < 0.001 each, R² < 0.1 each). All linear-regression correlations of cytokines in lung ADC are presented in Supplementary Figure 4. All linear-regression correlations of cytokines in lung SqCC are presented in Supplementary Figure 5.

**Figure 3:**
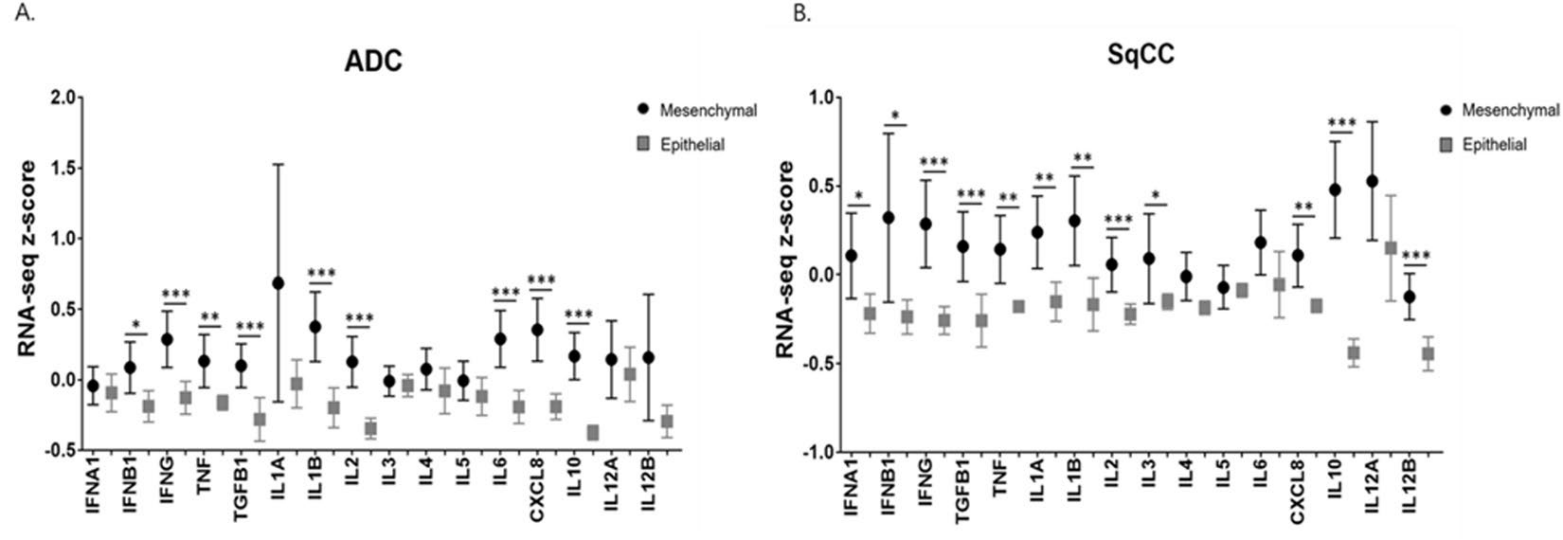
Box plot of RNA-seq z-scores of 16 cytokine genes for ‘mesenchymal’ and ‘epithelial’ groups. (**A**) Distribution of RNA-seq z-scores of ‘mesenchymal’ lung ADC compared to ‘epithelial’ lung ADC (**B**) Distribution of RNA-seq z-scores of ‘mesenchymal’ lung SqCC compared to ‘epithelial’ lung SqCC *p < *0.05*, **p < *0.01*, ***p < *0.001*.

Upon analysis, expression of the cytokines *IFNB1, IFNG (IFN-γ)*, *TNF (TNF-α), TGFB1* (*TGF-β), IL1B*, *IL2*, *IL6, CXCL8 (IL-8)*, and *IL10* were all significantly increased in ‘mesenchymal’ lung ADC compared to ‘epithelial’ tumors (Fig. 3A). In lung SqCC, expression of *IFNA1, IFNB1, IFNG (IFN-γ), TNF (TNF-α), TGFB1* (*TGF-β), IL1A, IL1B, IL2, IL3, CXCL8 (IL-8), IL10*, and *IL12B* all showed significantly increased z-score distribution in ‘mesenchymal’ lung SqCC (Fig. 3B). There was largely a trend of increased expression of all cytokines in ‘mesenchymal’ groups compared to ‘epithelial’ groups of NSCLCs.

### ‘Mesenchymal’ lung ADC and SqCC both showed significantly higher expression of immune checkpoint genes, with the exception of *PD-L1*

**Figure 4:**
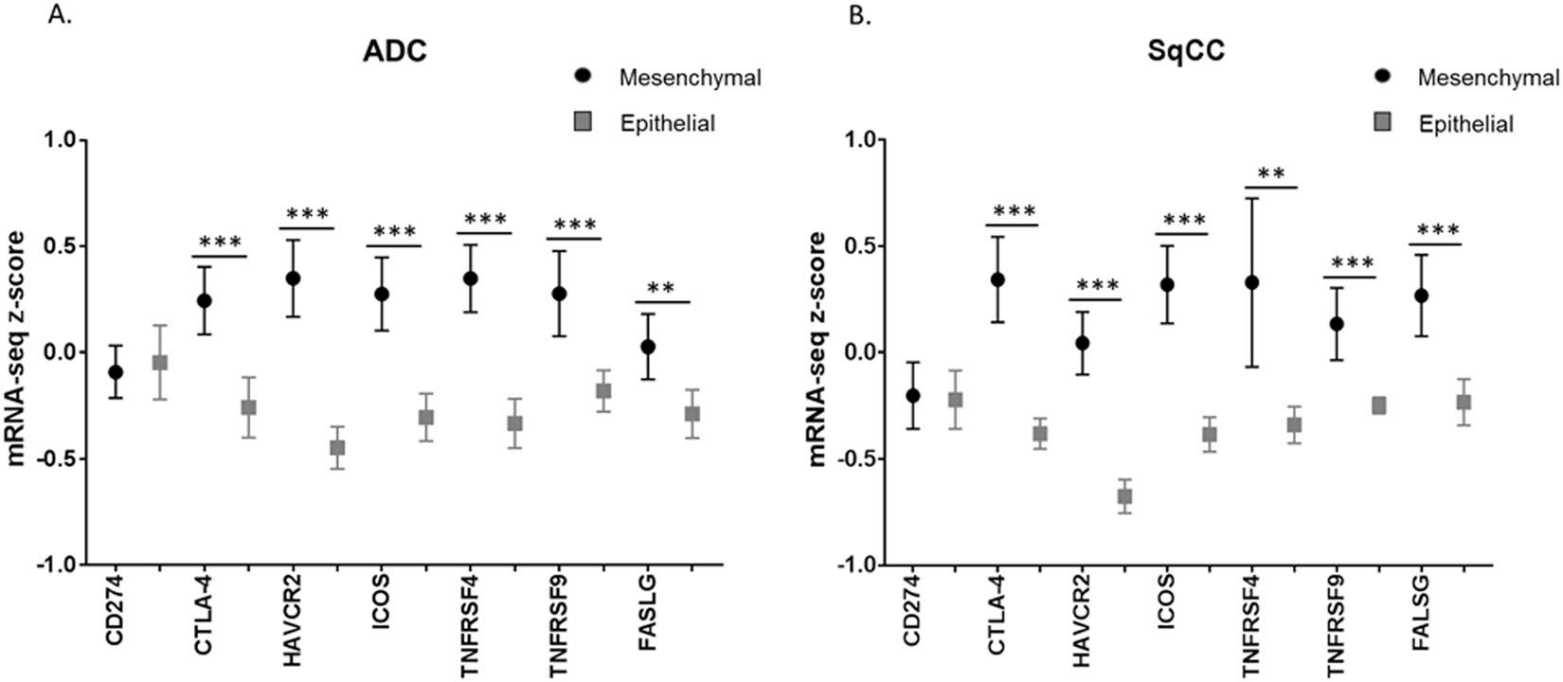
Box plot of RNA-seq z-scores of 6 immune checkpoint genes and *FASLG* for ‘mesenchymal’ and ‘epithelial’ groups. (**A**) Distribution of RNA-seq z-scores of ‘mesenchymal’ lung ADC compared to ‘epithelial’ lung ADC (**B**) Distribution of RNA-seq z-scores of ‘mesenchymal’ lung SqCC compared to ‘epithelial’ lung SqCC *p < *0.05*, **p < *0.01*, ***p < *0.001*.

Similar to our cytokine analysis, we analyzed the expression of the six immune checkpoint markers between ‘mesenchymal’ and ‘epithelial’ groups. In both lung ADC and SqCC, the ‘mesenchymal’ group showed increased expression of all immune checkpoint markers except for *PD-L1 (CD274)*. In addition, expression of *FASLG (FasL/CD95L)* was also significantly increased in both ‘mesenchymal’ NSCLCs (Fig. 4). Linear-regression analysis of all immune checkpoint markers, except for *CD274* in both lung ADC and SqCC showed a significantly positive correlation with high EMT score, while *FASLG (FasL/CD95L)* expression also showed a significantly positive correlation with EMT score in lung ADC and SqCC. All linear-regression correlations for lung ADC are presented in Supplementary Figure 6. All linear-regression correlations of lung SqCC are presented in Supplementary Figure 7.

Expression of epithelial genes *CDH1, DSP*, and *TJP1* showed a general negative correlation with expression of immune checkpoint genes and *FASLG*, while mesenchymal genes *VIM* and *SNAI1* showed a general positive correlation with expression of immune checkpoint genes and *FASLG* in both lung ADC and SqCC. The mesenchymal gene *CDH2* however did not show any correlation with expression of immune checkpoint genes and *FASLG*. All linear-regression correlations for lung ADC are presented in Supplementary Figure 8. All linear-regression correlations of lung SqCC are presented in Supplementary Figure 9.

### Overexpression of individual mesenchymal genes was associated with significantly lower infiltration of activated CD4/CD8 T-cells in lung ADC and SqCC patients

To determine which individual ‘mesenchymal’ and ‘epithelial’ EMT marker genes were associated with immune exclusion, we studied the immune landscape of each individual gene separately. Each individual ‘mesenchymal’ gene that significantly decreased infiltration levels (p < 0.05) of both activated CD4 and CD8 T-cells in high expression groups were deemed meaningful. *VIM* showed significantly decreased infiltration of activated CD4 and CD8 T-cells (p < 0.001 each), as well as mDC, TGD, and Th17 cells (p < 0.001 each) in the high expression group while the infiltration of macrophages and Treg cells were significantly increased (p < 0.001 each). Likewise, five other genes showed a significantly decreased infiltration of both activated CD4/CD8 T-cells (*FOXC2*; p < 0.001/p = 0.012, *ITGB6*; p < 0.001 each, *MMP2*; p < 0.001 each, *MMP9*; p = 0.016/p < 0.001, and *SOX10*; p < 0.001 each) in high expression groups. In lung SqCC, 9 ‘mesenchymal’ genes including *VIM* showed significantly decreased infiltration of both activated CD4/CD8 T-cells in high expression groups (*VIM*; p < 0.001 each, *CDH2*; p = 0.003/p < 0.001, *FOXC2*; p < 0.001 each, *SNA1*; p < 0.001 each, *FN1*; p < 0.001 each, *ITGB6*; p < 0.001 each, *MMP2*; p < 0.001 each, *MMP3*; p < 0.001 each, and *MMP9*; p < 0.001 each).

For ‘epithelial’ genes, high expression group of *DSP* showed significantly increased infiltration of activated CD4/CD8 T-cells in lung ADC (p < 0.001 each). In lung SqCC, significantly increased infiltration was seen only for activated CD4 T-cells in high expression group of *DSP* (p = 0.0164). Though not statistically significant, activated CD8 T-cells also showed a trend towards increased infiltration (p = 0.1799). *TJP1* showed significantly decreased infiltration of activated CD8 T-cells in high expression group (p < 0.001) of lung ADC, while the level of activated CD4 T-cells was similar in high and low expression groups. In lung SqCC, infiltration of activated CD4 T-cells was increased in the high expression group of *TJP1*, though not significantly (p = 0.3418), while infiltration of activated CD8 T-cells was significantly decreased (p < 0.001). The immune infiltration and overall immune landscape of 31 immune cells for each gene for lung ADC and lung SqCC are presented in Supplementary Figure 10, and Supplementary Figure 11, respectively.

### Infiltration of CD8 T-cells did not affect the OS between ‘mesenchymal’ and ‘epithelial’ groups in both lung ADC and SqCC

There was no appreciable difference in OS between ‘mesenchymal’ and ‘epithelial’ groups for both ADC and SqCC (p = 0.9086, p = 0.1293 respectively) (Supplementary Figure 12A).

Furthermore, infiltration of activated CD8 T-cells did not affect OS in ‘mesenchymal’ and ‘epithelial’ groups. There was no significant difference in OS between the four groups in either lung ADC or SqCC (p = 0.3872, p = 0.2353 respectively) (Supplementary Figure 12B).

## Discussion

We comprehensively analyzed the TME and immune landscape of the infiltration of 31 immune cells in both lung ADC and SqCC^24^. As EMT has been associated with immune exclusion from the TME, we hypothesized that ‘mesenchymal’ NSCLCs would display decreased infiltration by antitumor immune cell types. In line with our expectations, activated CD4 and CD8 T-cells, as well as mDCs, all showed relatively decreased infiltration in ‘mesenchymal’ lung ADC and SqCC. CD4 and CD8 T-cells play critical roles in immunosurveillance against cancer while mDCs confer an increase in immune activation by antigen presentation and activation of those T-cells^26^. While the infiltration of immune cells with antitumor functions were decreased, infiltration of immune cells with immunosuppressive functions were increased in ‘mesenchymal’ NSCLCs. Activated B cell infiltration was significantly increased in both NSCLCs, while Treg infiltration was significantly increased in SqCC and showed a trending increased infiltration in ADC. Although the function of B cells in cancer is not completely understood, they have been shown to secrete immunosuppressive cytokines such as IL-10, IL-4, and TGF-β in cancer^27^, where IL-10 and TGF-β are able to inhibit the cytotoxic activities of CD8 T-cells^28^. Similarly, Tregs promote tumor progression in a number of ways, including inhibiting the activation and survival of T-cells and increasing levels of IL-10 and TGF-β^29–31^.

Interestingly, our results showed significantly increased infiltration of TGD in ‘mesenchymal’ lung ADC and SqCC, while infiltration of Th17 cells were significantly decreased in both ‘mesenchymal’ groups. TGDs have dual opposing functions in protection against cancer and promotion of tumor growth^32^. While they have highly effective tumor killing abilities, they are also known to promote tumor growth via IL-17 production. IL-17 is a pleiotropic cytokine which has shown increased expression levels in lung cancer that may also have a role in promoting EMT^33^. Th17 immune cells are defined by their production of IL-17, and along the same lines as TGD, also have dual opposing functions as they acquire either immune suppressive functions or antitumor functions depending on the tumor type and stage of progression^3,34^. Although TGD and Th17 both have potentially pro and antitumor functions, it is unclear which of the two functionalities were at play in ‘mesenchymal’ NSCLCs.

In recent years, inflammation has been established as a key inducer of EMT during the progression of cancer^35^. Modification of the TME during EMT occurs as a result of the activity of cytokines, such as IFN-*γ*, TGF-β and TNF-α which have been shown to induce EMT during cancer progression^31,36^. Our data corroborates previous findings of an inflammatory TME associated with EMT, as ‘mesenchymal’ lung ADC and SqCC tumors displayed a trend towards increased expression of all cytokines and growth factor genes compared to ‘epithelial’ NSCLCs. Expression of EMT inducing cytokines *IFN-γ* and *TNF-α* were both significantly increased in ‘mesenchymal’ groups while expression of immunosuppressive cytokines *IL-10* and *TGF-β* were also significantly increased in both ‘mesenchymal’ NSCLCs. TGF-β is not only immunosuppressive but as previously mentioned, also has EMT inducing and T-cell inhibiting functions. Furthermore, pro-inflammatory cytokines such as IL-1, IL-6, and IL-8 which induce chronic inflammation may lead to transformation of cells and malignancy^31,37^. In line with such findings, our results showed significantly increased expression of *IL1B*, *IL6*, and *CXCL8 (IL-8)* in ‘mesenchymal’ ADC, while *IL1A*, *IL1B*, and *CXCL8 (IL-8)* showed significantly increased expression in ‘mesenchymal’ SqCC. Finally, expression of *IL2*, which is known to be critical for the development and peripheral expansion of Tregs^38^ was significantly increased in both NSCLCs.

While it has been shown that *PD-L1* expression levels in ‘mesenchymal’ lung ADC were significantly elevated^18^, we did not observe any difference in RNA expression levels of *PD-L1* between ‘mesenchymal’ and ‘epithelial’ groups in the two NSCLCs. Although the aforementioned study used an expanded 76 gene marker for EMT score, it showed a significant correlation to the EMT score derived from the 16 canonical gene markers utilized in our study (r value of 0.58 with a p value ~0 in a Pearson’s correlation)^21^. As such, *PD-L1* is expected to show higher expression levels in ‘mesenchymal’ ‘groups of NSCLC. However, as our results did not show a difference between the two groups, it suggests that the markers utilized in the two studies may not be completely accurate.

Other immune inhibitory checkpoint genes, *CTLA-4* and *HAVCR2* (*TIM-3)* were significantly overexpressed in ‘mesenchymal’ lung ADC and SqCC, in addition to *FASLG (FasL/CD95L)* which has both immunosuppressive and antiangiogenic effects^39^. Furthermore, immune stimulatory checkpoint genes^40–42^, namely *ICOS*, *TNFRSF4 (OX40/CD134)*, and *TNFRSF9* (*4-1BB/CD137)* were also all overexpressed in ‘mesenchymal’ NSCLCs. This is in agreement with previous results speculating that an immunosuppressive TME led to functional impairment of T-cells following initial proper activation^18^. This, along with the overexpression of immunosuppressive cytokines, suggests that the TME in ‘mesenchymal’ NSCLCs is in a state of overall immune suppression. Although analysis using the EMT score did show a correlation with increased expression of inflammatory cytokines and immune checkpoint genes, correlation results of analysis at the individual gene level were unclear. If at all, the correlation was very weak, although statistically significant. Nonetheless, the directions of correlation for ‘mesenchymal’ and ‘epithelial’ genes were consistent with our hypothesis as ‘epithelial’ gene markers of EMT showed a general negative correlation with expression of immune checkpoint genes while the opposite was shown for ‘mesenchymal’ gene markers of EMT. The caveat is that the study of individual gene expression of factors affecting the TME in relation to mRNA expression of individual EMT signature genes alone does have its inherent limitations, compared to analysis with the EMT score.

Although features of EMT are associated with progression of cancer^43^, ‘mesenchymal’ lung ADC and SqCC did not show any significant differences in OS. This was consistent with a prior study which showed that EMT status and poorer survival were not correlated in cancers, including lung cancer^44^. Furthermore, we, for the first time, looked at whether the presence of infiltrating activated CD8 T-cells altered OS between ‘mesenchymal’ and ‘epithelial’ groups. Although infiltration of lymphocytes has been correlated with improved outcome^5,7^, our results did not show a difference between the two groups. This may be attributed to the fact that tumor samples in TCGA are generally not of advanced stage patients^45^, and the role of CD8 T-cells in advanced and early stage cancers may be different.

Our findings of infiltration of immune cells and expression of various cytokines, growth factors, and immune checkpoints indicate a TME where antitumor function of T-cells are largely inhibited in ‘mesenchymal’ lung ADC and SqCC. While the associations and specific mechanisms are as of yet unclear, what is clear is that a complex interplay exists between the dynamic process that is EMT and the TME, cytokines, and tumor-infiltrating immune cells. Though we can infer that an association is present between these factors and EMT, our results do have limitations. As this is a study of an RNA-seq clinical database, our findings need to be validated at the protein level in future studies. Another shortcoming of this study is that TCGA tumor samples are core biopsies or incisional biopsies, which may not be a reliable representative of the entire tumor^46^.

In summary, ‘mesenchymal’ features of lung ADC and SqCC are associated with exclusion of activated T-cells in the TME, which in turn may be associated with an upregulation of inflammatory cytokines in the TME as well as an upregulation of immunosuppressive immune checkpoint factors. As has been shown in our study as well as previous studies, inflammatory cytokines may play an important role in the induction of EMT. In addition, there appears to be manipulation of immune checkpoints leading to an immunosuppressive TME. However, the interaction between the TME, cytokines, immune checkpoints, and immune cells are complex and their mechanisms and specific associations are ultimately poorly understood. To date, no clear causality between immune exclusion and EMT has been revealed in NSCLC. Its identification could ultimately play a valuable role in elucidating causes for resistance to immunotherapy, and therefore warrants further investigation.

## Electronic supplementary material

Supplementary Information
