# Supplementary material for: Epithelial-mesenchymal transition (EMT) signature is inversely associated with T-cell infiltration in non-small cell lung cancer (NSCLC)
Source: Sci Rep. 2018 Feb 13;8:2918. doi: 10.1038/s41598-018-21061-1 (PMC5811447; doi:10.1038/s41598-018-21061-1)
Supplement: Supplementary file 1 — Supplementary Information [file 41598_2018_21061_MOESM1_ESM.pdf]

Epithelial-mesenchymal transition (EMT) signature is inversely associated with T-cell infiltration in non-small cell lung cancer (NSCLC)

Young Kwang Chae[1*], Sangmin Chang[1*], Taeyeong Ko[1], Jonathan Anker[1], Sarita Agte[1], Wade Iams[1], Wooyoung M. Choi[1], Kyoungmin Lee[2], Marcelo Cruz[1]

[1] Northwestern University Feinberg School of Medicine, Department of Medicine, Chicago, IL

[2] Department of Internal Medicine, Asan Medical Center, University of Ulsan College of Medicine, Seoul, Republic of Korea

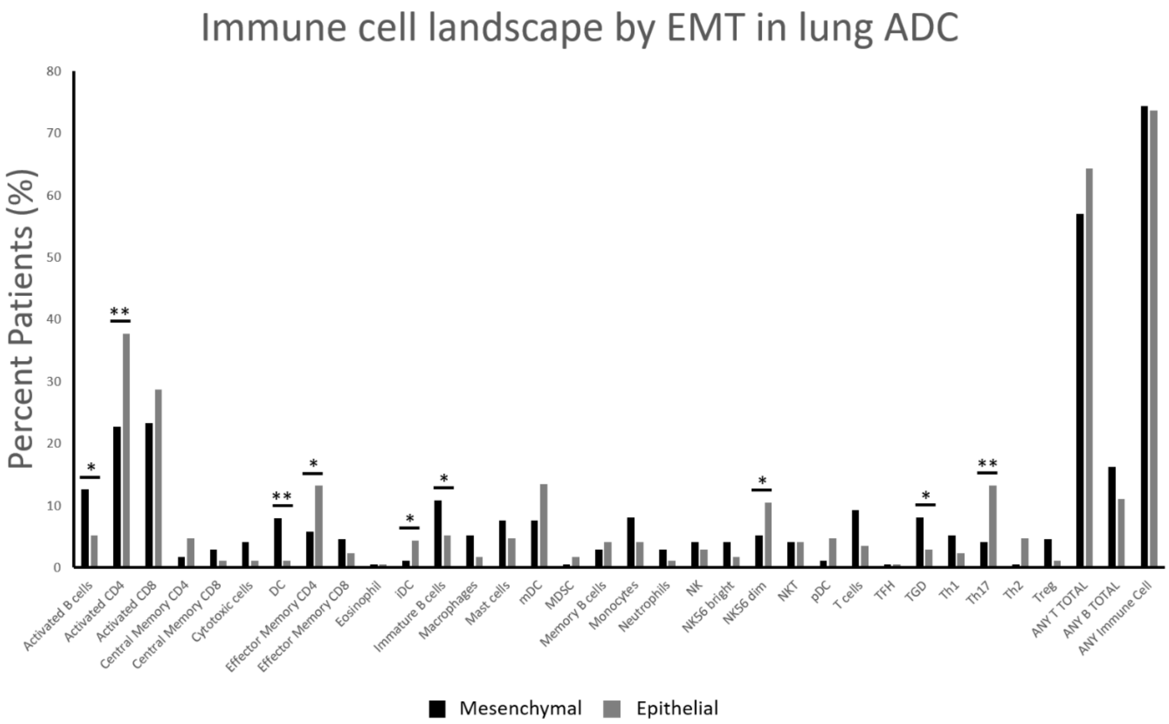

Supplementary Figure 1. Immune cell infiltration landscape by EMT score status. Immune infiltration of 31 distinct immune cells of ‘mesenchymal’ lung ADC compared to ‘epithelial’ lung ADC. * *p<0.05, *** *p<0.01, **** *p<0.001.*

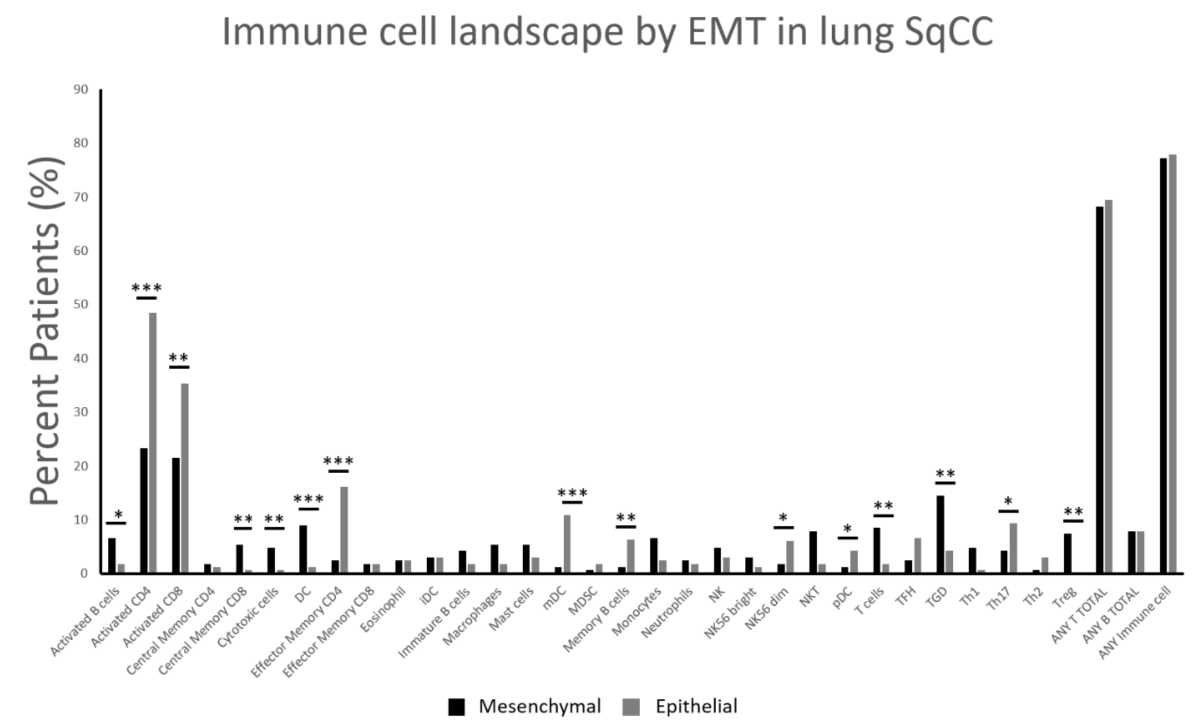

Supplementary Figure 2. Immune cell infiltration landscape by EMT score status. Immune infiltration of 31 distinct immune cells of 'mesenchymal' lung SqCC compared to 'epithelial' lung SqCC. * *p<0.05*, ** *p<0.01*, *** *p<0.001*.

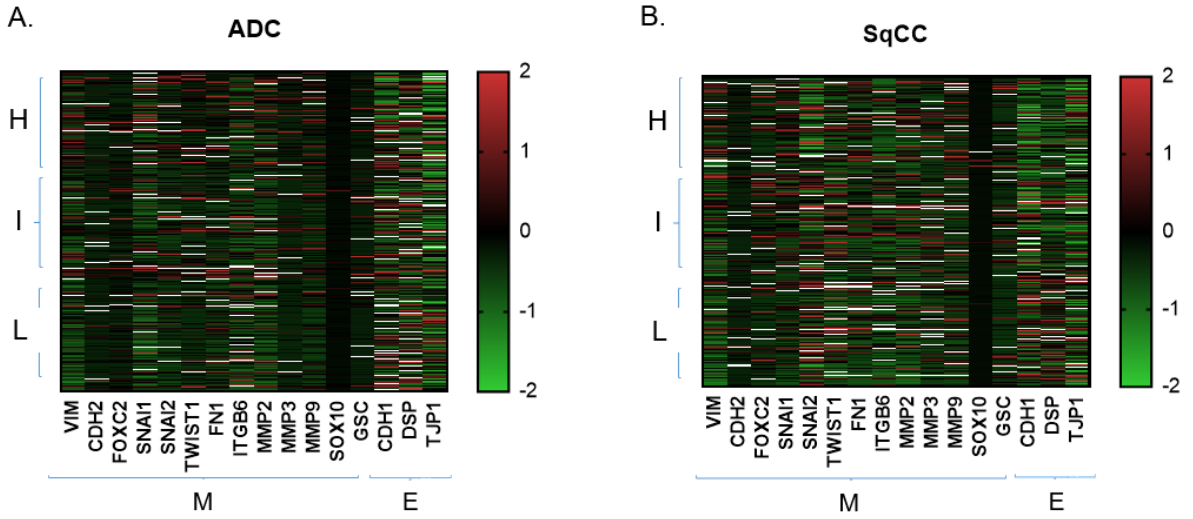

Supplementary Figure 3. A. Mesenchymal and epithelial gene expression in terms of CD8 T-cell signature score of lung ADC. **B.** Mesenchymal and epithelial gene expression in terms of CD8 T-cell signature score of lung SqCC. *H: high CD8 T-cell signature score, I: intermediate CD8 T-cell signature score, L: low CD8 T-cell signature score, M: mesenchymal genes, E: epithelial genes*

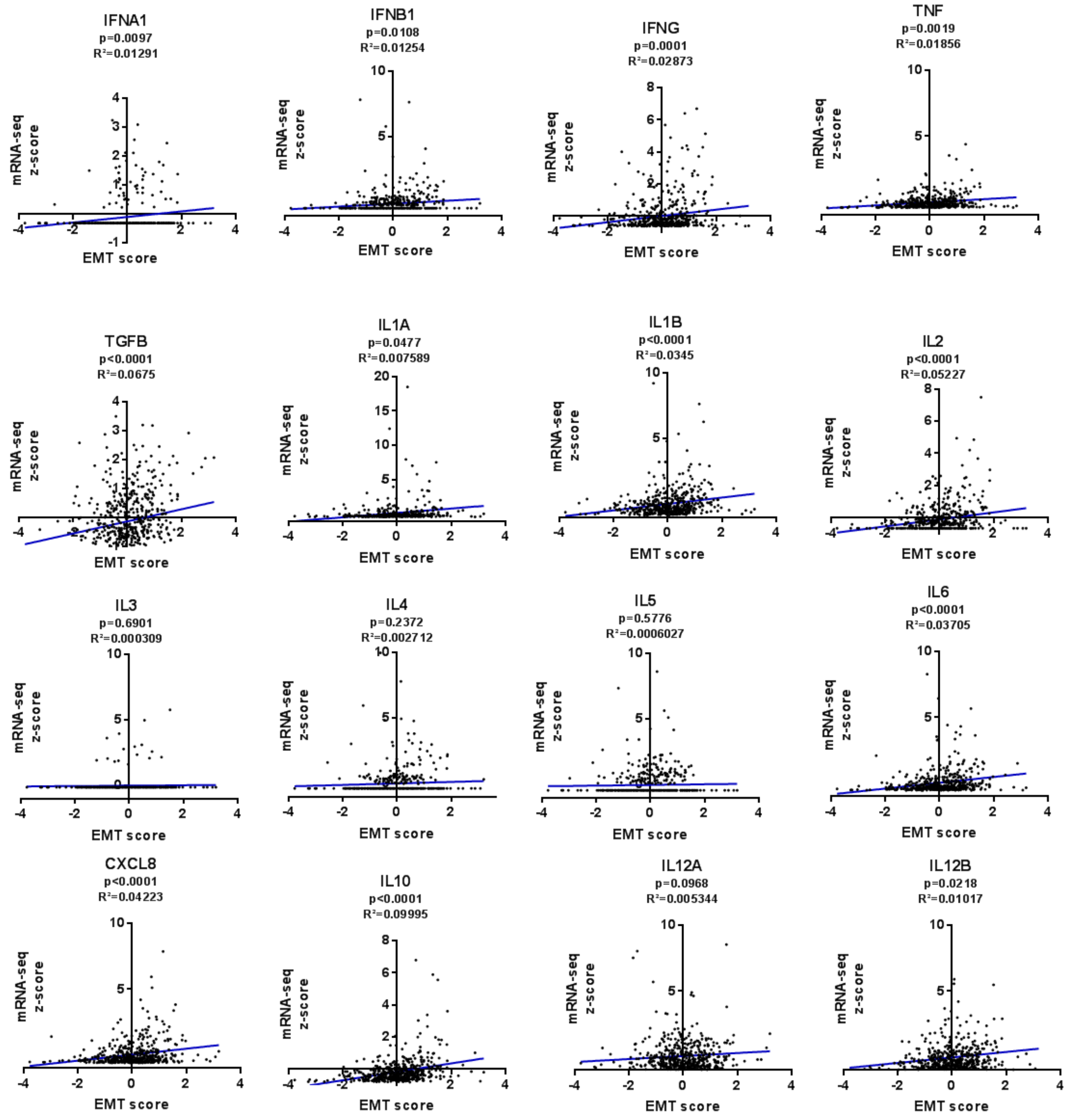

Supplementary Figure 4. Linear-regression correlation between mRNA-seq z-score expression of cytokines and EMT score in lung ADC.

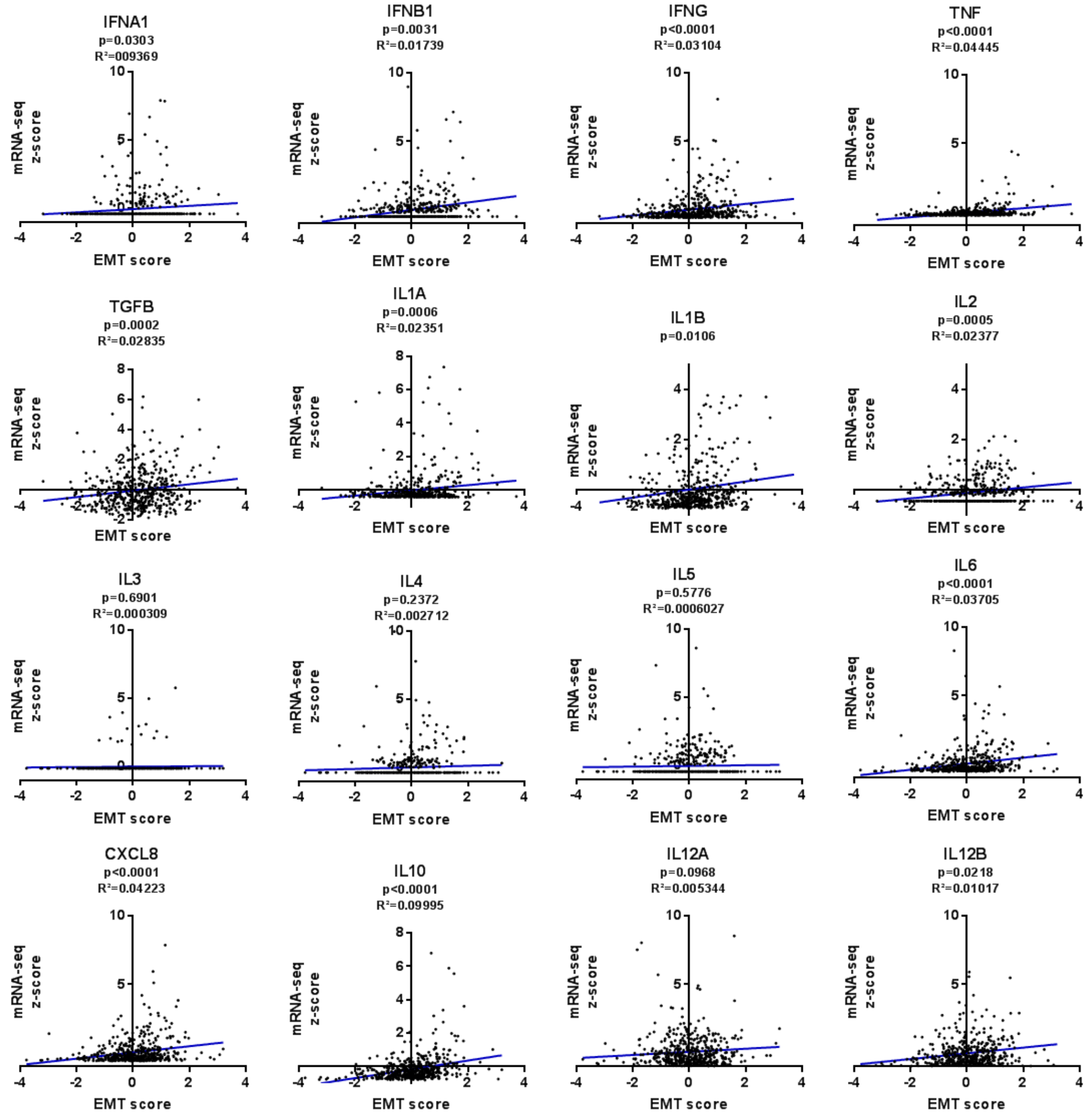

Supplementary Figure 5. Linear-regression correlation between mRNA-seq z-score expression of cytokines and EMT score in lung SqCC.

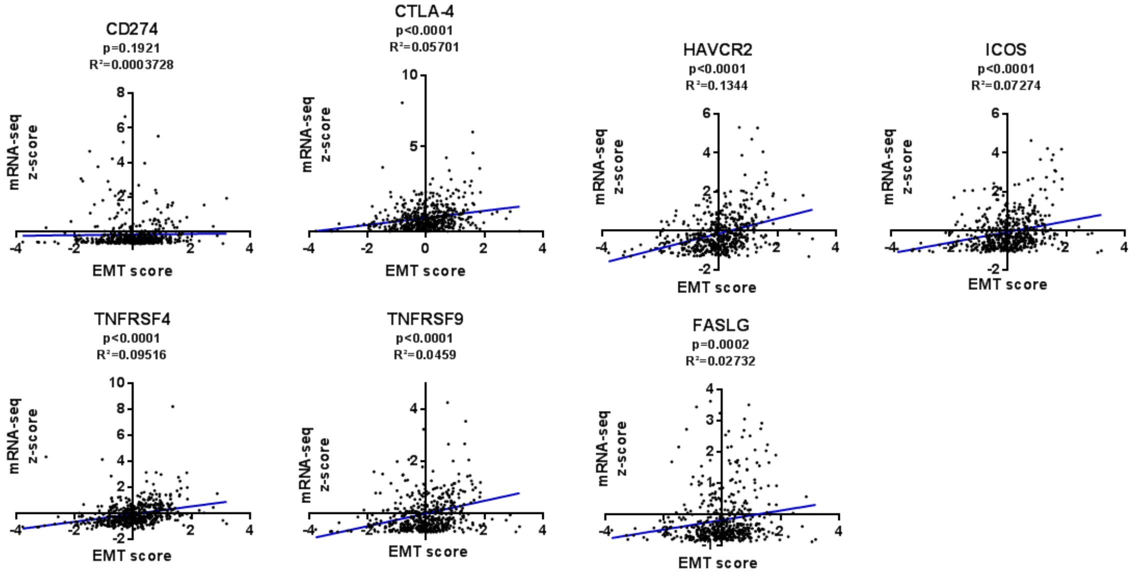

Supplementary Figure 6. Linear-regression correlation between mRNA-seq z-score expression of immune checkpoint markers and EMT score in lung ADC.

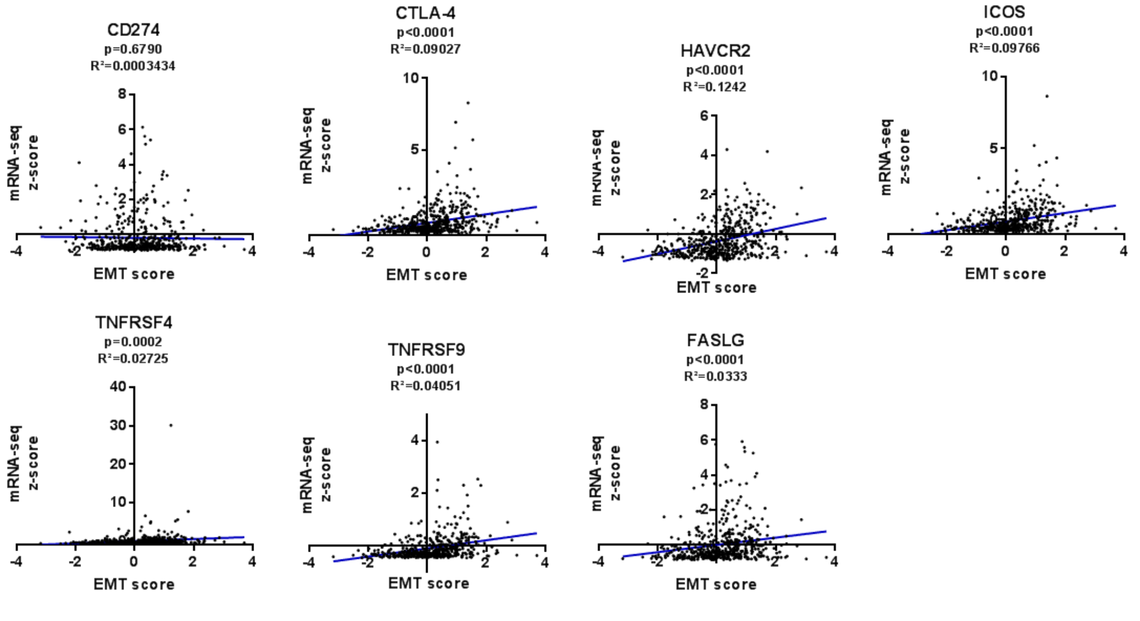

Supplementary Figure 7. Linear-regression correlation between mRNA-seq z-score expression of immune checkpoint markers and EMT score in lung SqCC.

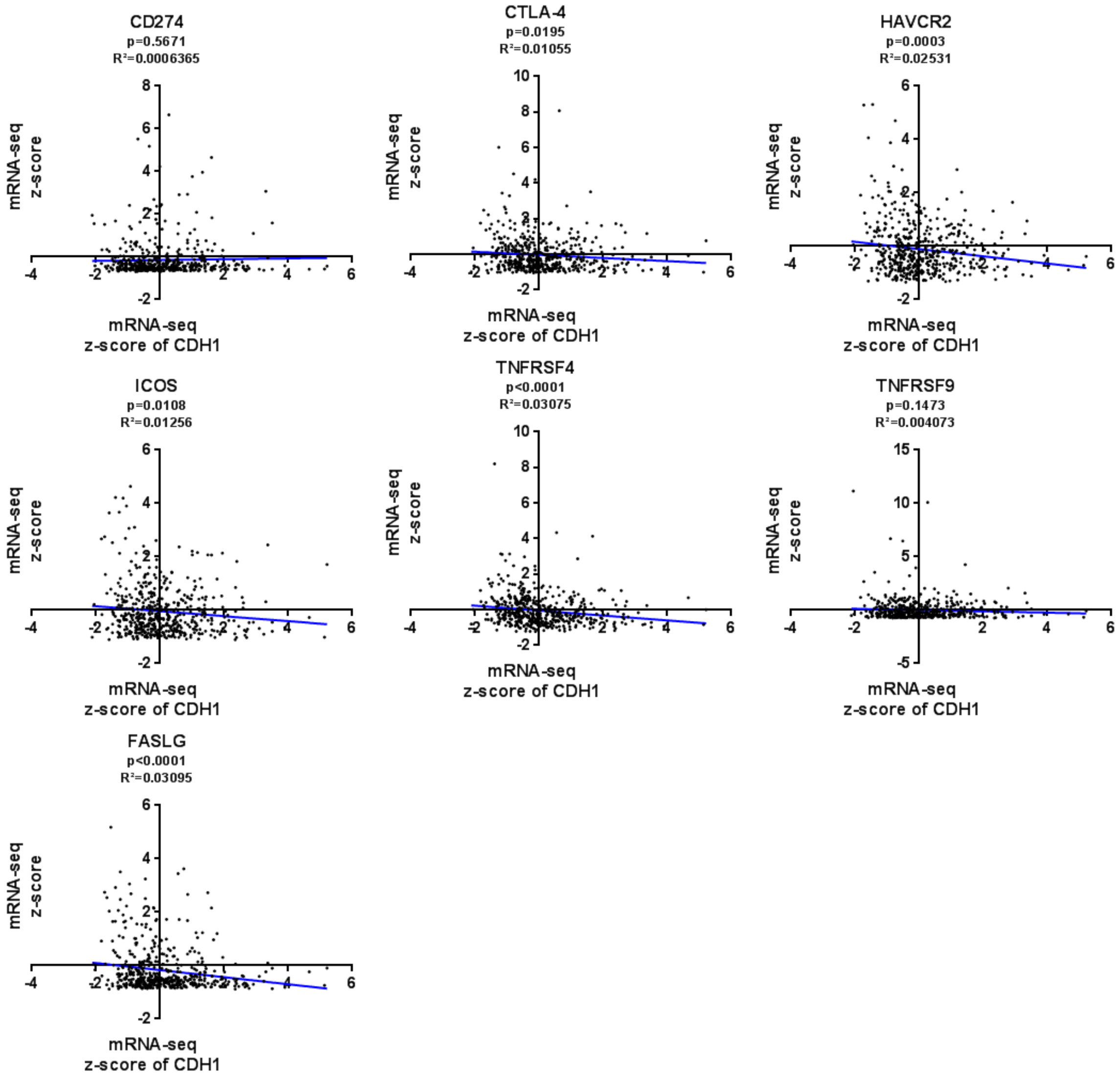

Supplementary Figure 8. A. Linear-regression correlation between mRNA-seq z-score expression of immune checkpoint markers and mRNA-seq z-score of epithelial gene marker *CDH1* in lung ADC.

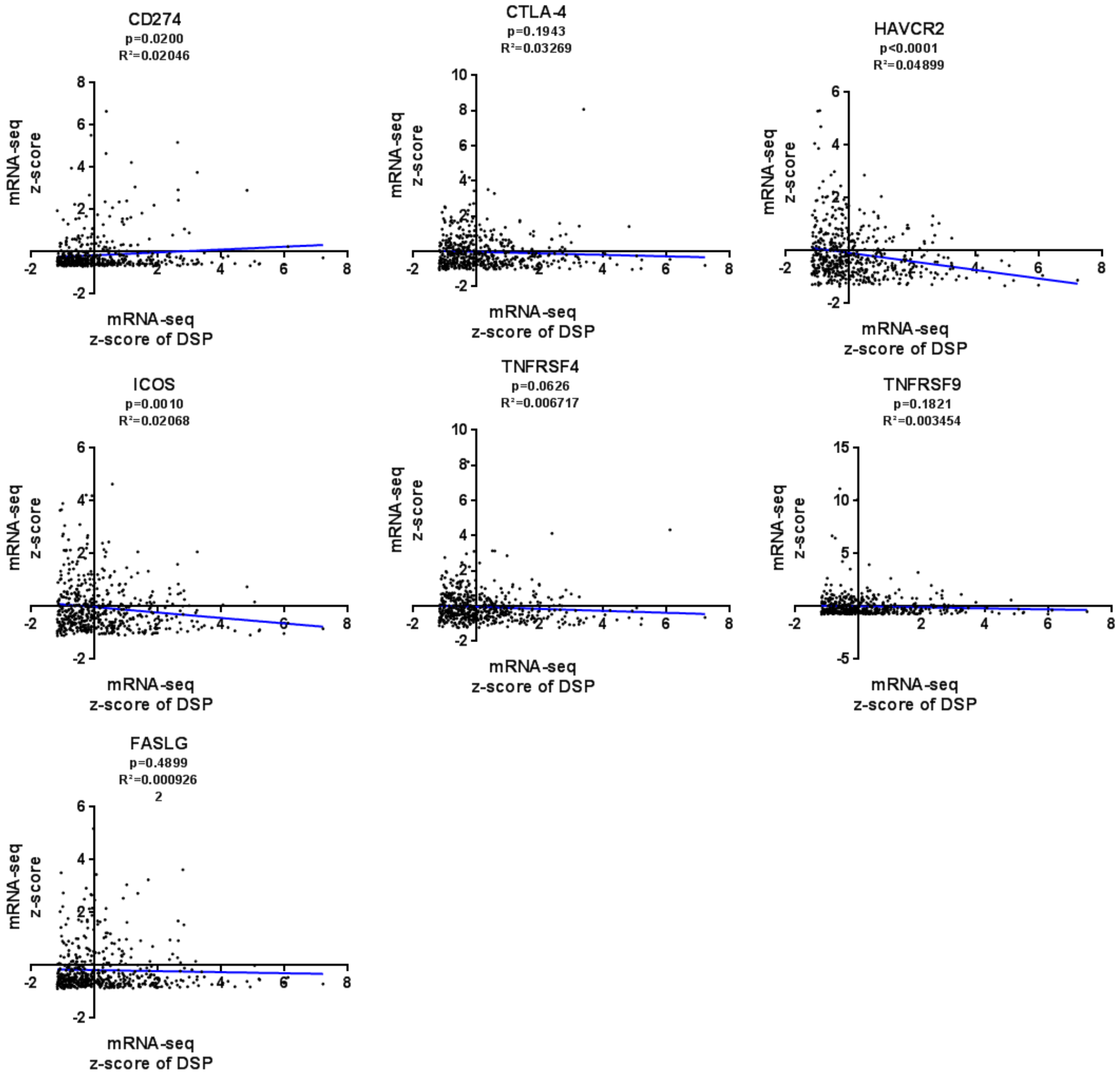

Supplementary Figure 8. B. Linear-regression correlation between mRNA-seq z-score expression of immune checkpoint markers and mRNA-seq z-score of epithelial gene marker *DSP* in lung ADC.

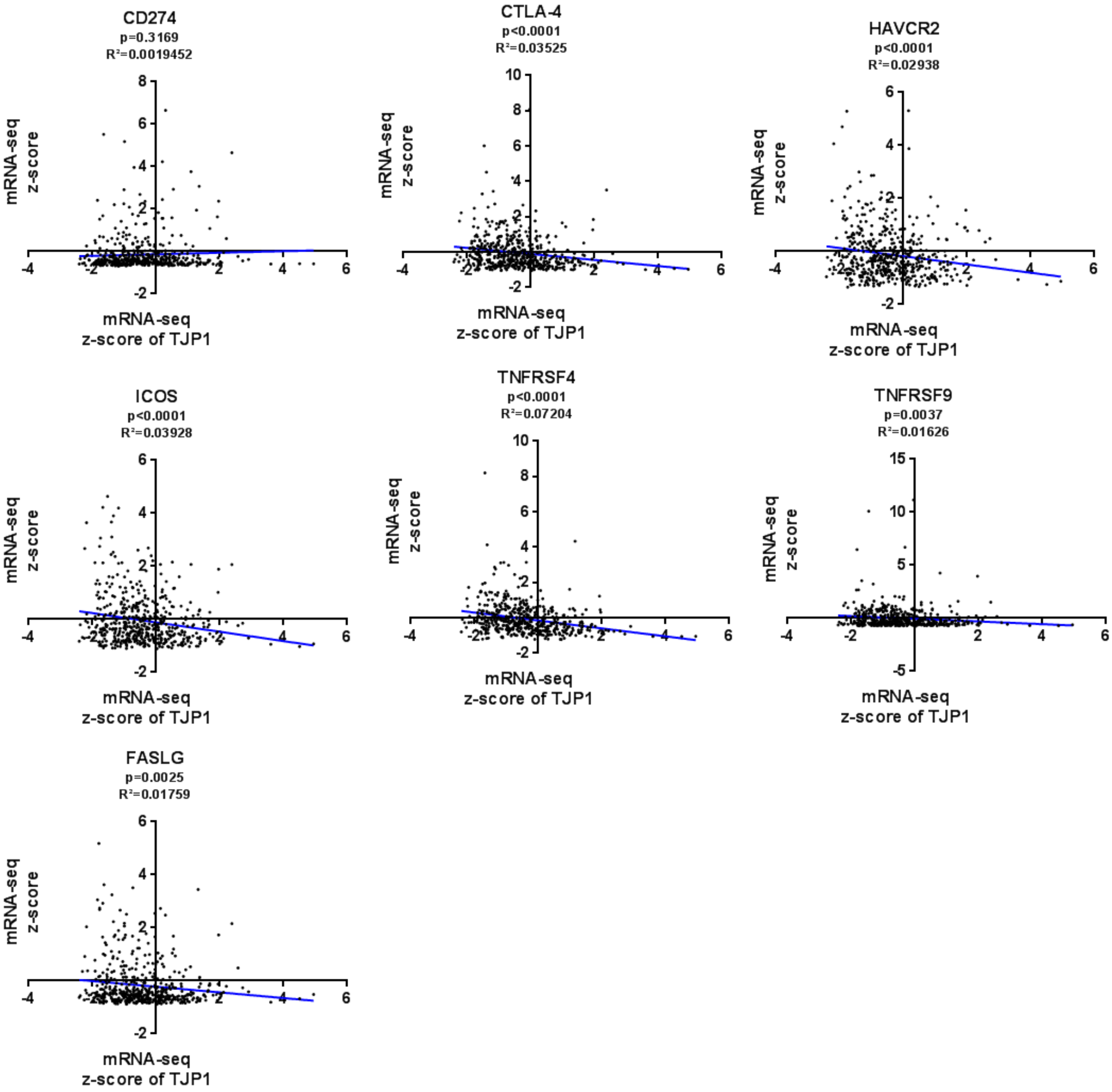

Supplementary Figure 8. C. Linear-regression correlation between mRNA-seq z-score expression of immune checkpoint markers and mRNA-seq z-score of epithelial gene marker *TJP1* in lung ADC.

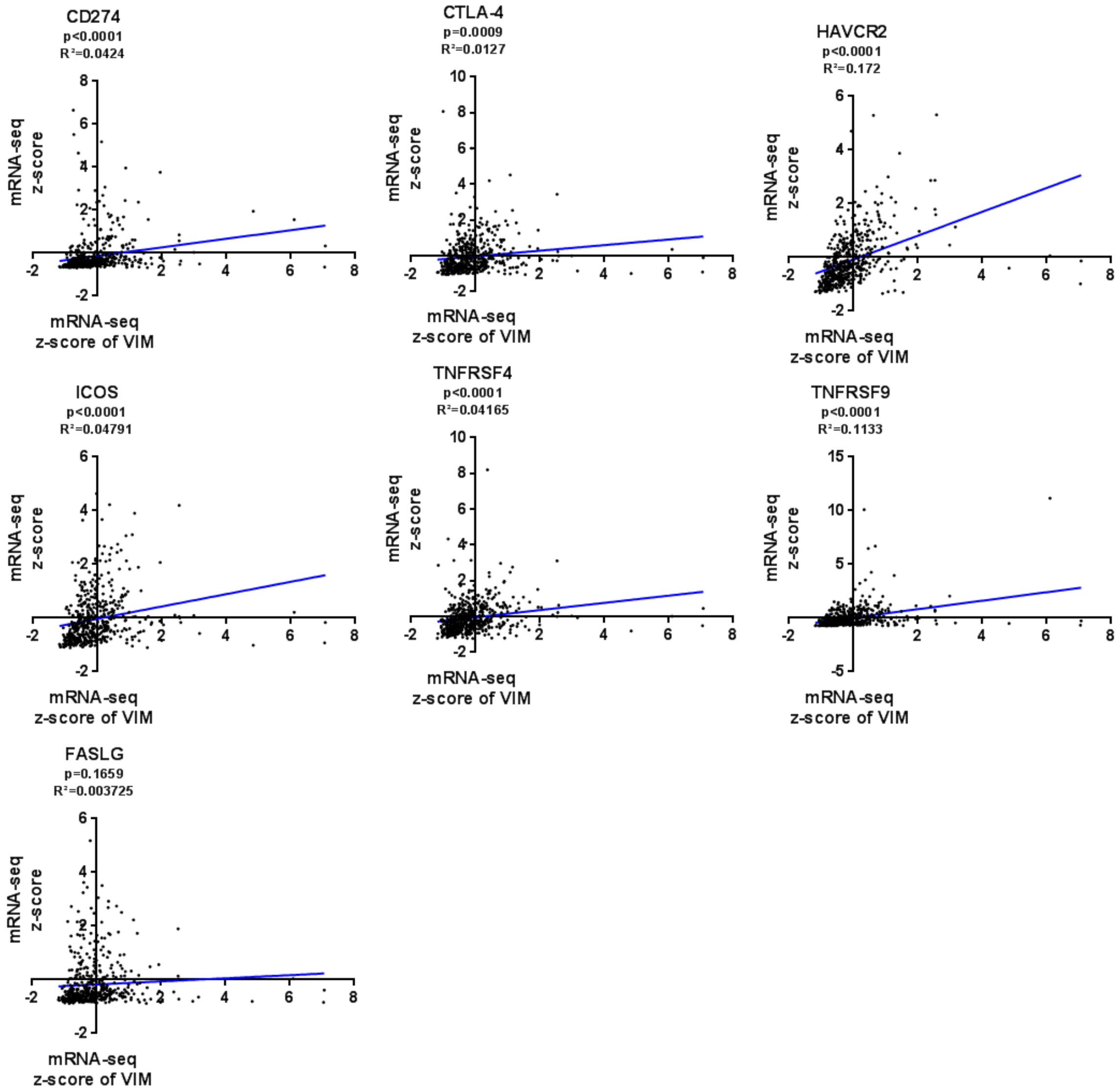

Supplementary Figure 8. D. Linear-regression correlation between mRNA-seq z-score expression of immune checkpoint markers and mRNA-seq z-score of mesenchymal gene marker *VIM* in lung ADC.

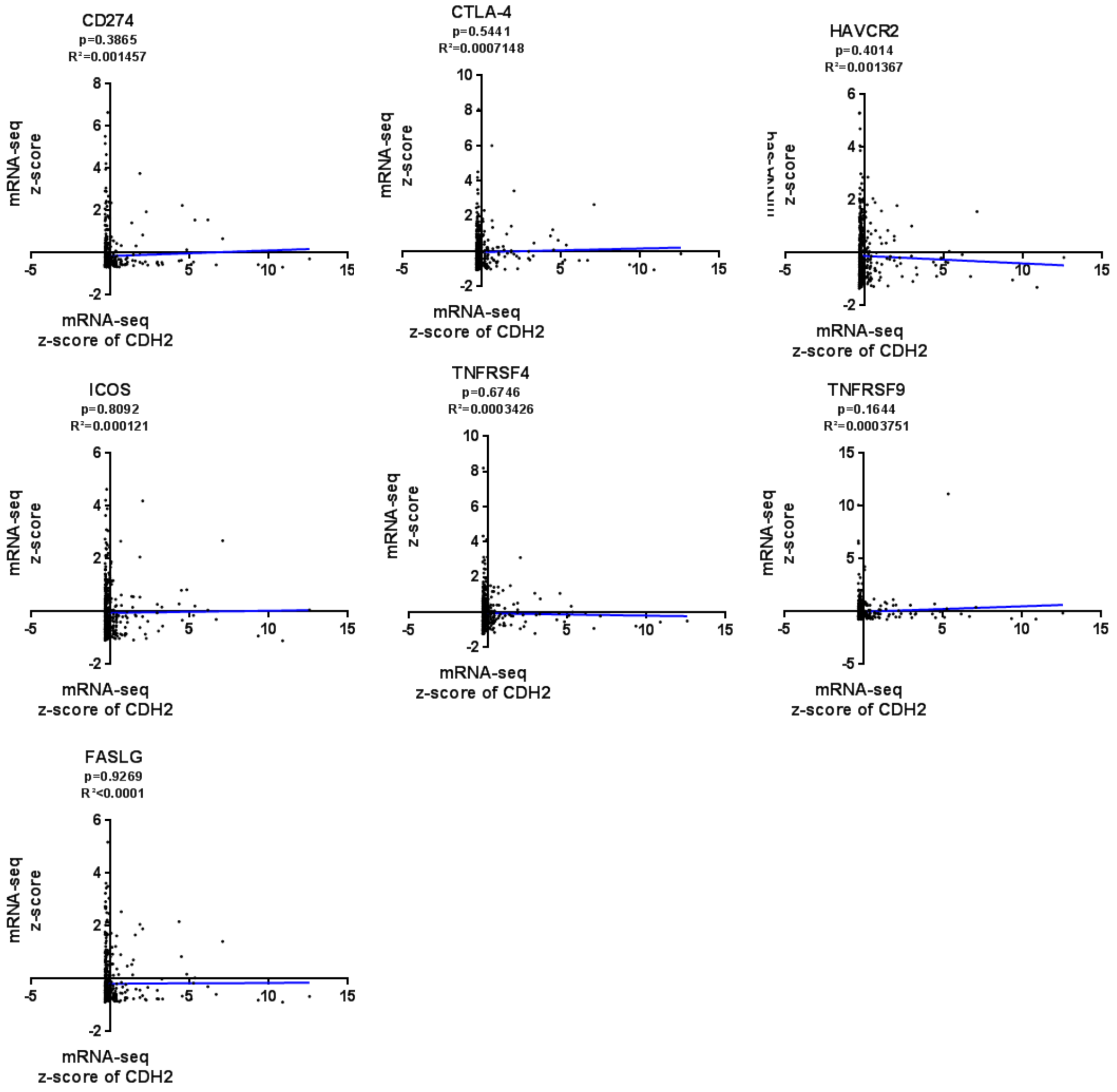

Supplementary Figure 8. E. Linear-regression correlation between mRNA-seq z-score expression of immune checkpoint markers and mRNA-seq z-score of mesenchymal gene marker *CDH2* in lung ADC.

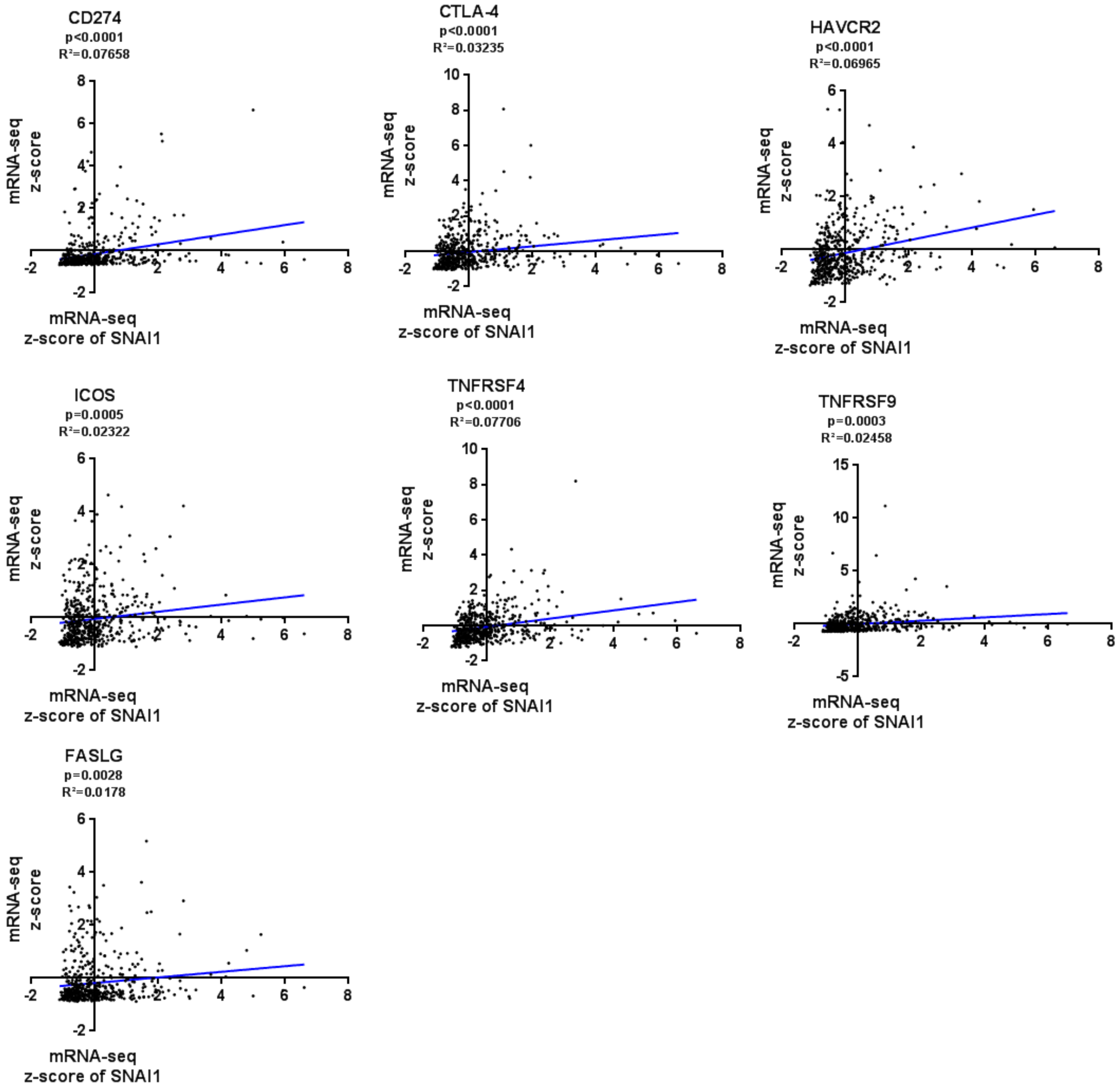

Supplementary Figure 8. E. Linear-regression correlation between mRNA-seq z-score expression of immune checkpoint markers and mRNA-seq z-score of mesenchymal gene marker *SNAI1* in lung ADC.

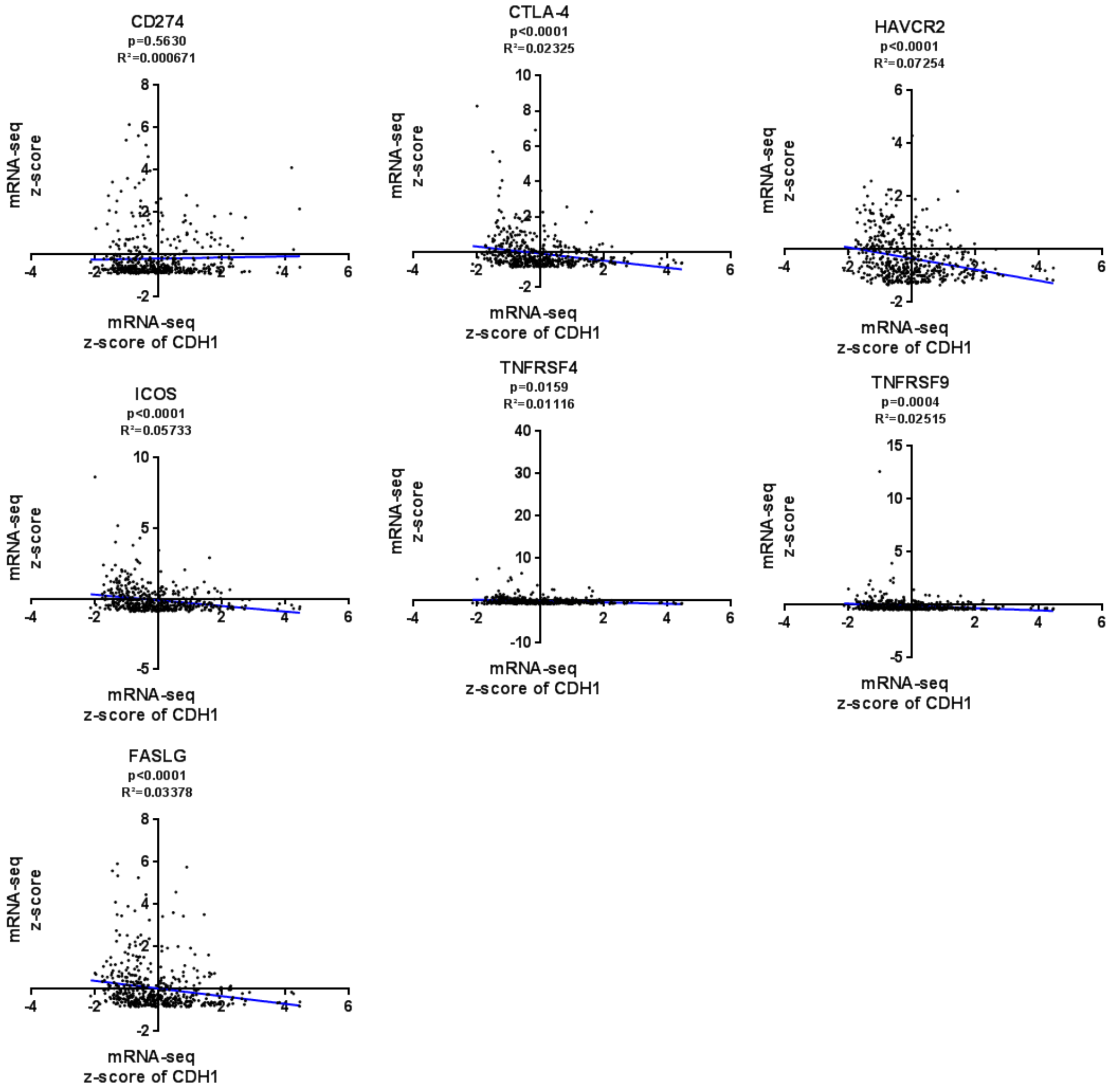

Supplementary Figure 9. A. Linear-regression correlation between mRNA-seq z-score expression of immune checkpoint markers and mRNA-seq z-score of epithelial gene marker *CDH1* in lung SqCC.

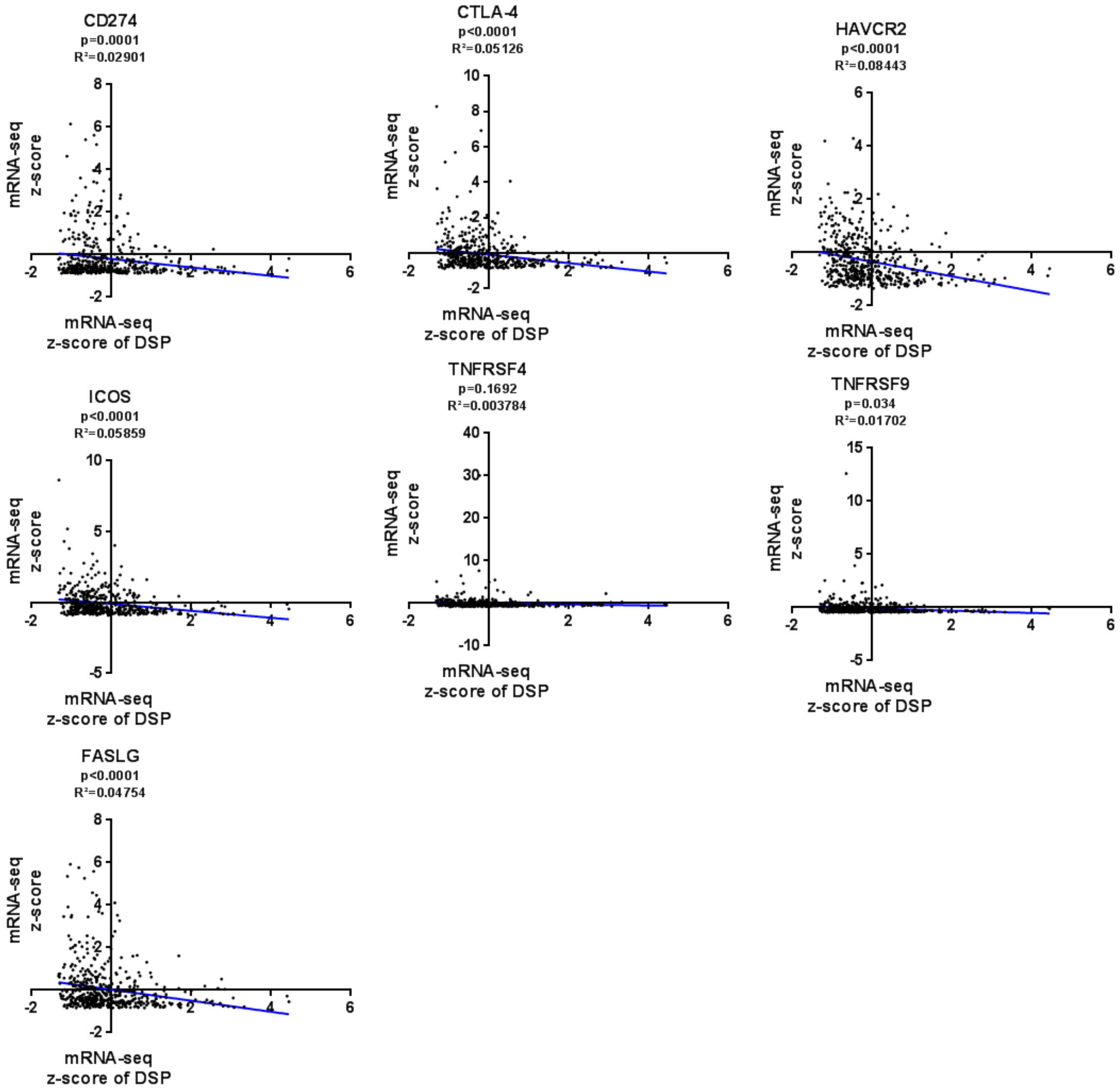

Supplementary Figure 9. B. Linear-regression correlation between mRNA-seq z-score expression of immune checkpoint markers and mRNA-seq z-score of epithelial gene marker *DSP* in lung SqCC.

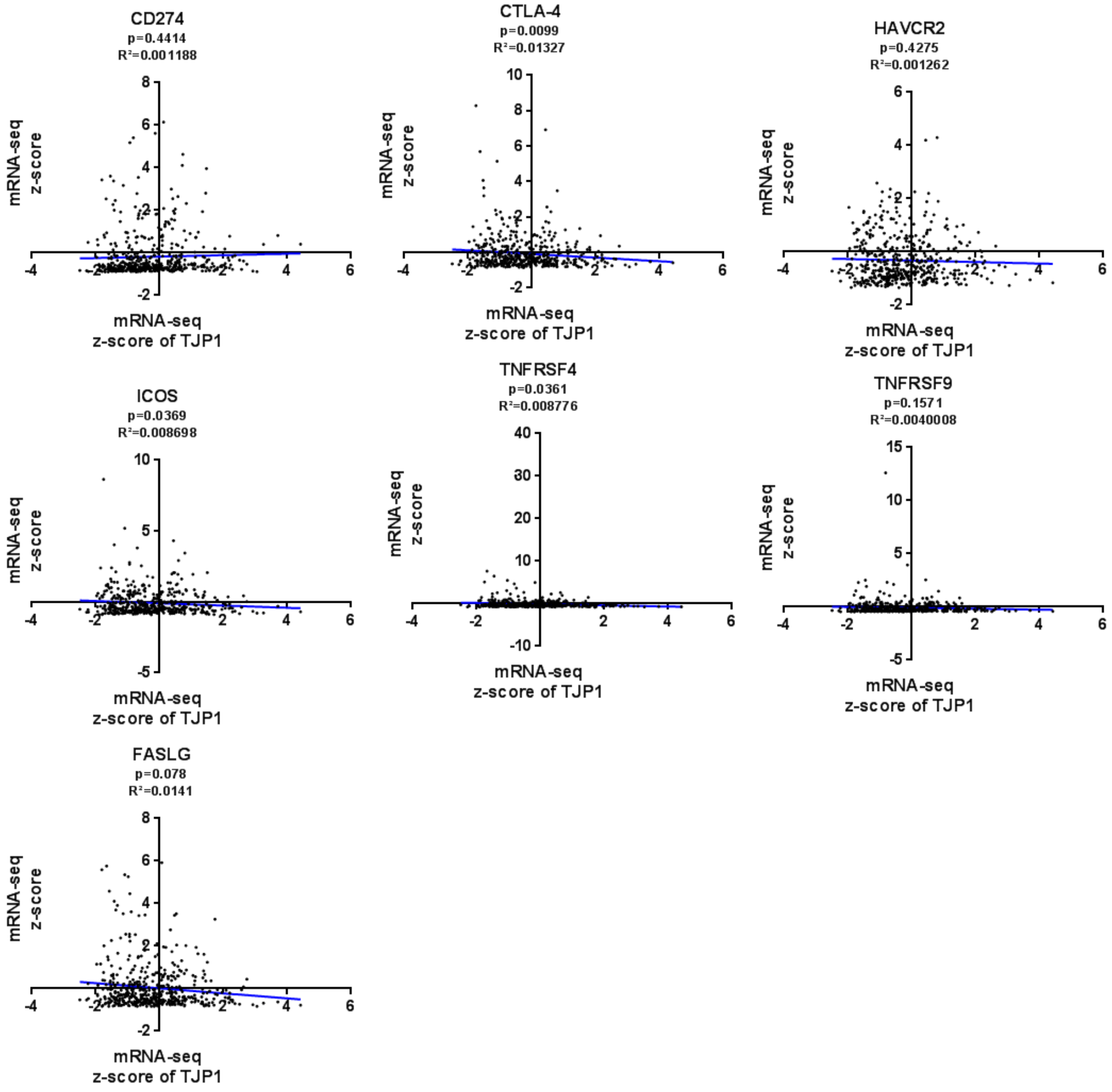

Supplementary Figure 9. C. Linear-regression correlation between mRNA-seq z-score expression of immune checkpoint markers and mRNA-seq z-score of epithelial gene marker *TJP1* in lung SqCC.

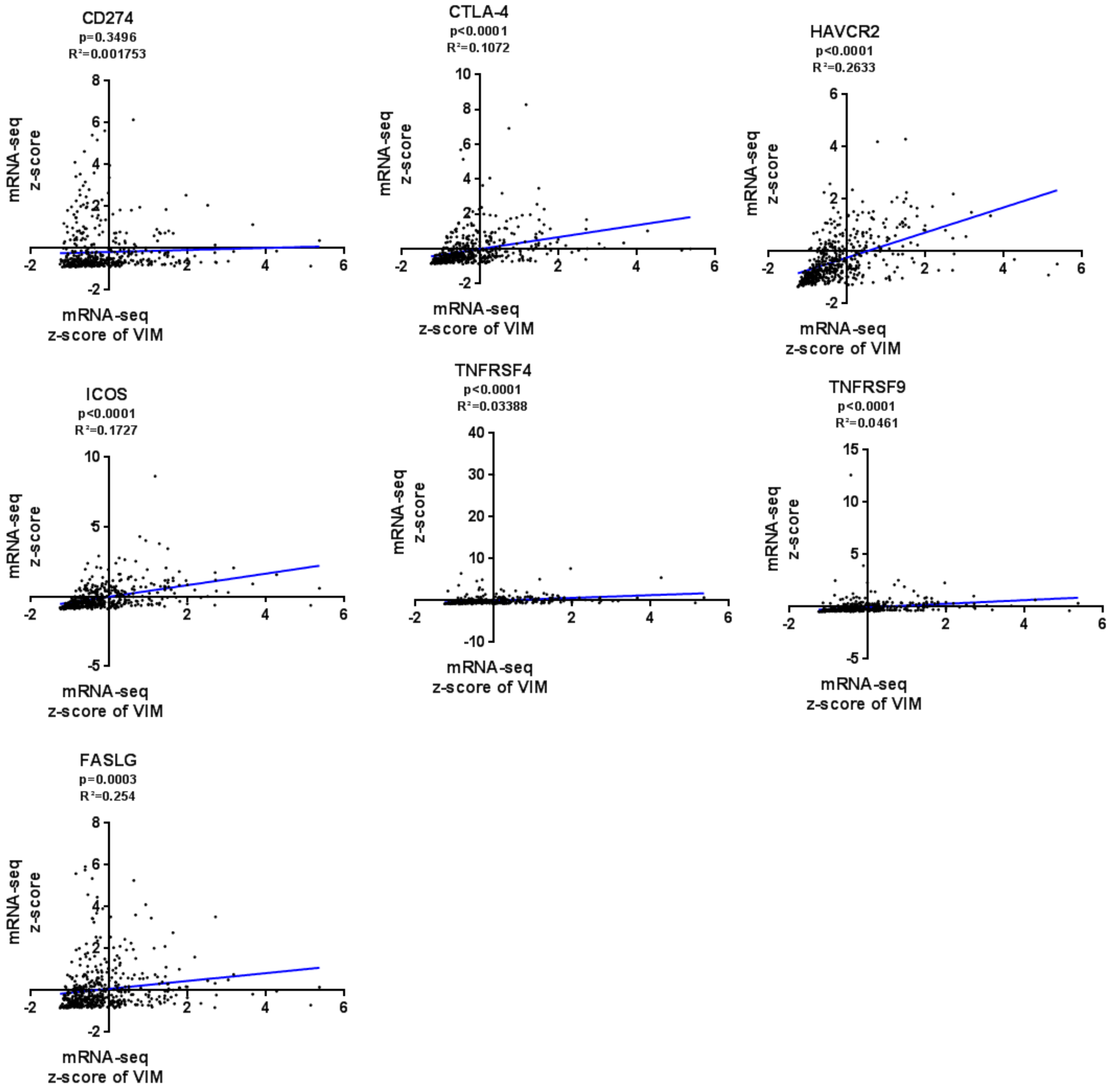

Supplementary Figure 9. D. Linear-regression correlation between mRNA-seq z-score expression of immune checkpoint markers and mRNA-seq z-score of mesenchymal gene marker *VIM* in lung SqCC.

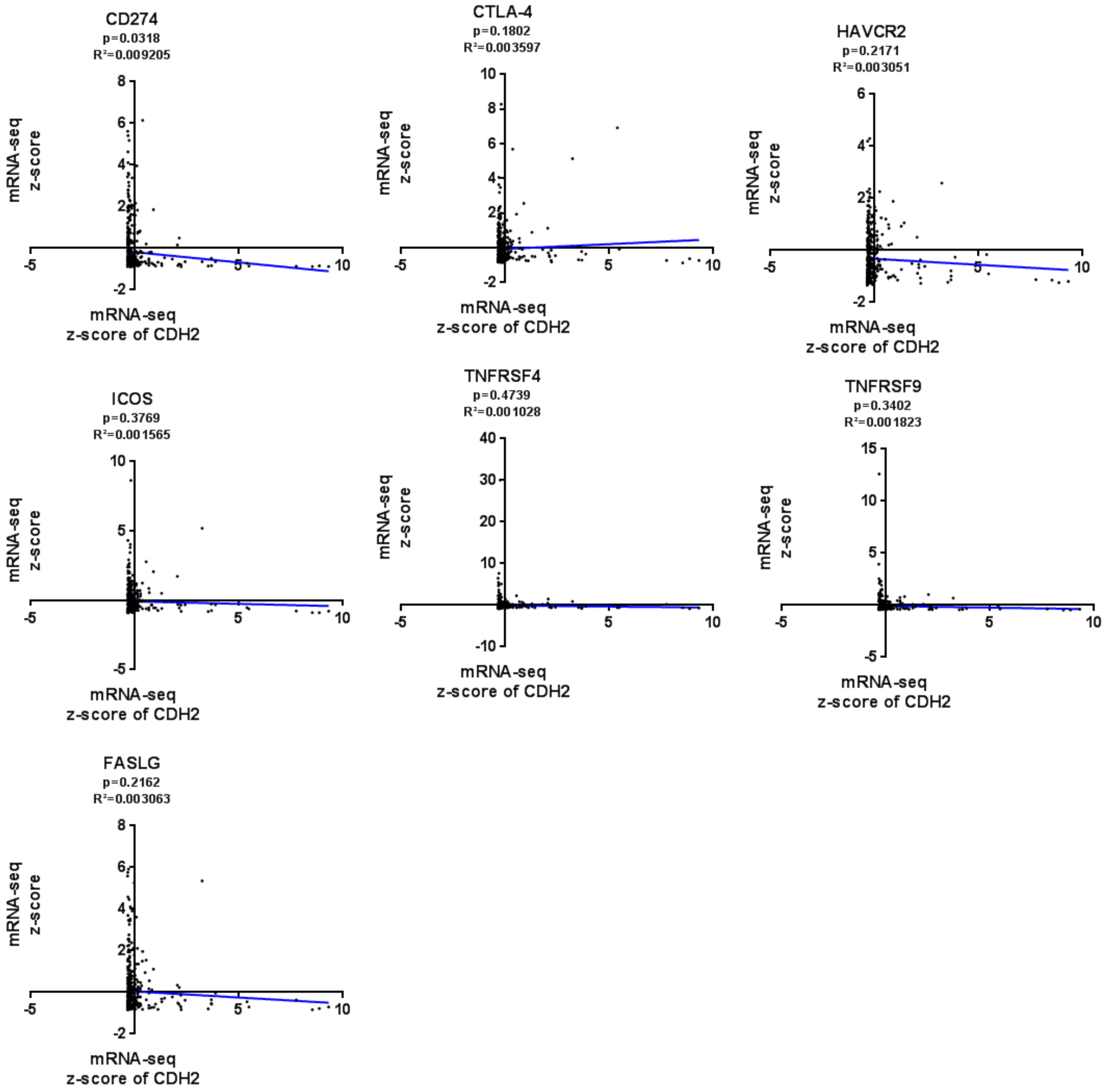

Supplementary Figure 9. E. Linear-regression correlation between mRNA-seq z-score expression of immune checkpoint markers and mRNA-seq z-score of mesenchymal gene marker *CDH2* in lung SqCC.

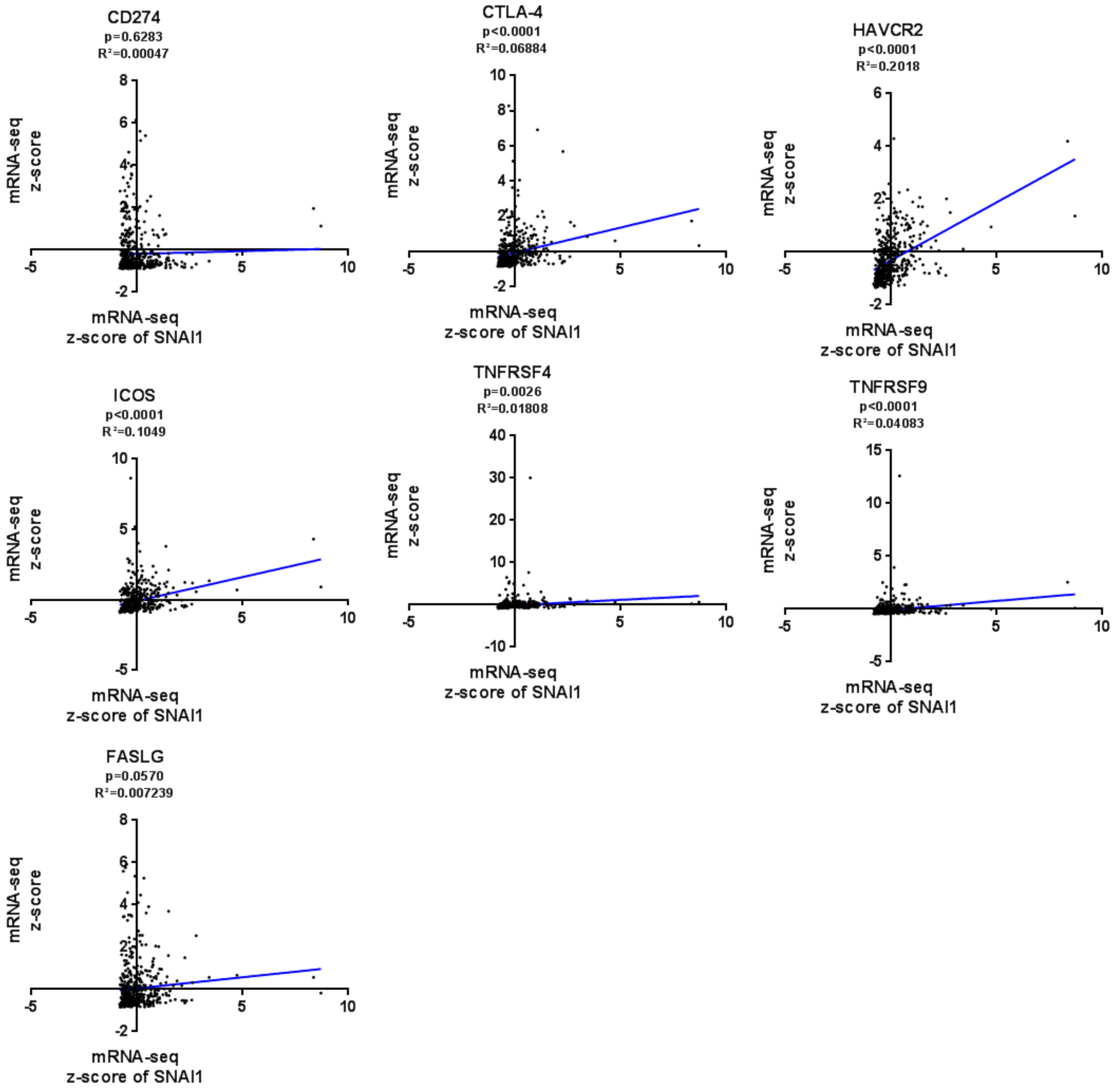

Supplementary Figure 9. F. Linear-regression correlation between mRNA-seq z-score expression of immune checkpoint markers and mRNA-seq z-score of mesenchymal gene marker *SNAI1* in lung SqCC.

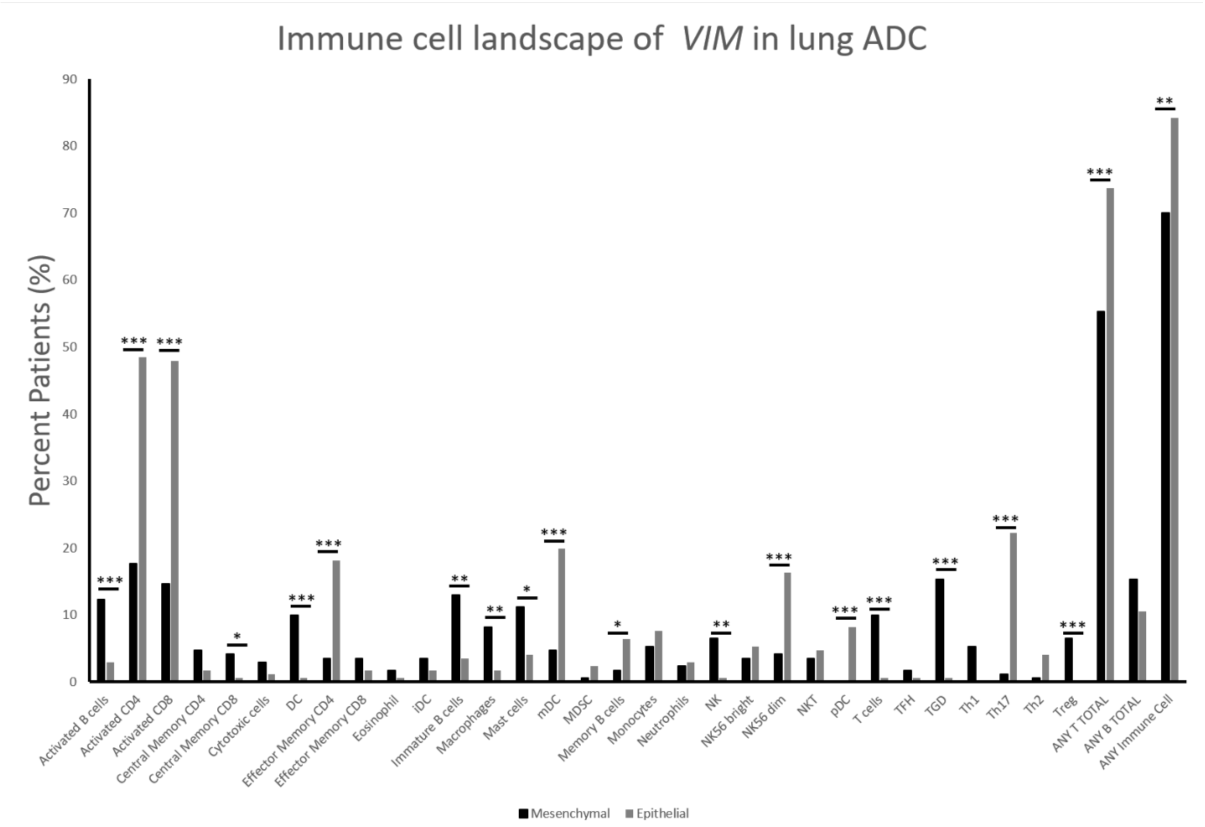

Supplementary Figure 10. A. Immune cell infiltration landscape of *VIM* in lung ADC by EMT score status. Immune infiltration of 31 distinct immune cells of 'mesenchymal' lung ADC compared to 'epithelial' lung ADC. ** p<0.05, ** p<0.01, *** p<0.001.*

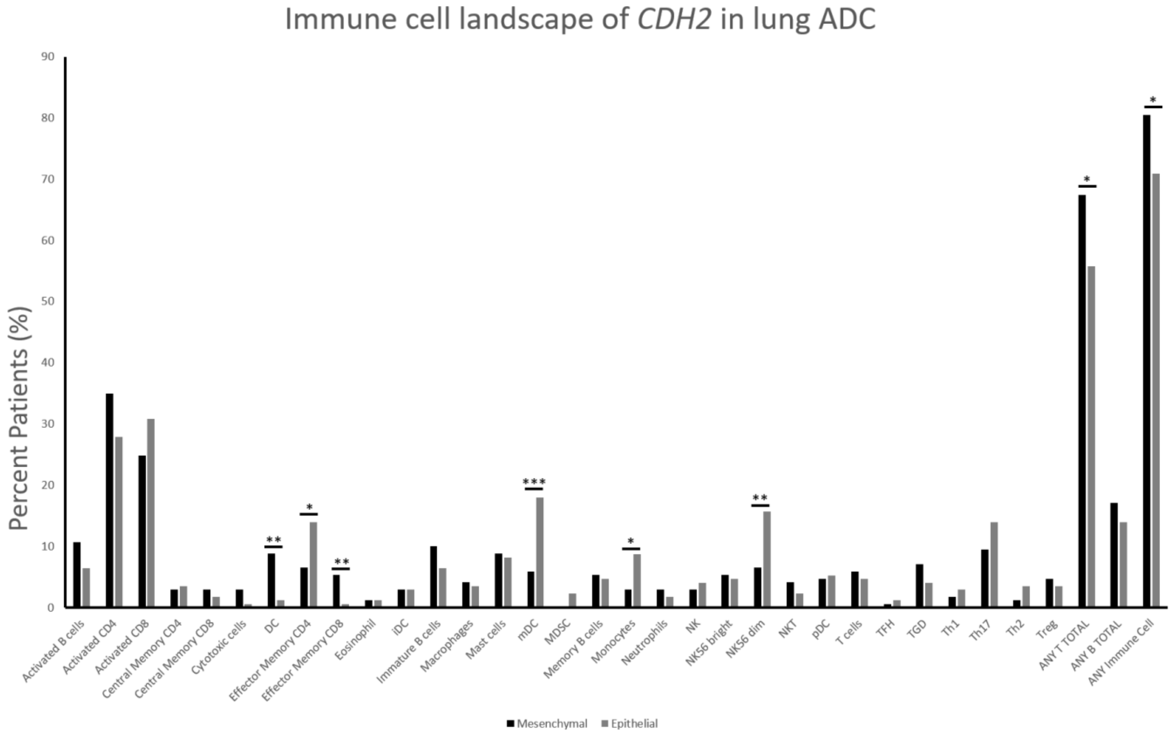

Supplementary Figure 10. B. Immune cell infiltration landscape of *CDH2* in lung ADC by EMT score status. Immune infiltration of 31 distinct immune cells of ‘mesenchymal’ lung ADC compared to ‘epithelial’ lung ADC. * $p<0.05$, ** $p<0.01$, *** $p<0.001$.

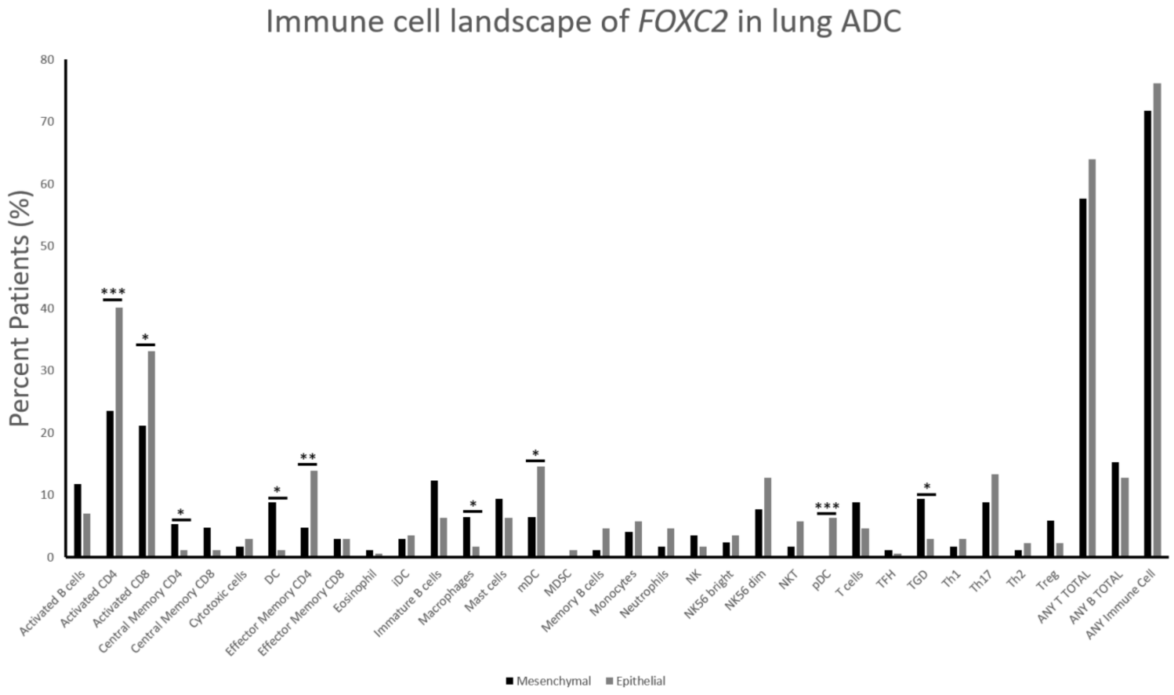

Supplementary Figure 10. C. Immune cell infiltration landscape of *FOXC2* in lung ADC by EMT score status. Immune infiltration of 31 distinct immune cells of 'mesenchymal' lung ADC compared to 'epithelial' lung ADC. * *p<0.05*, ** *p<0.01*, *** *p<0.001*.

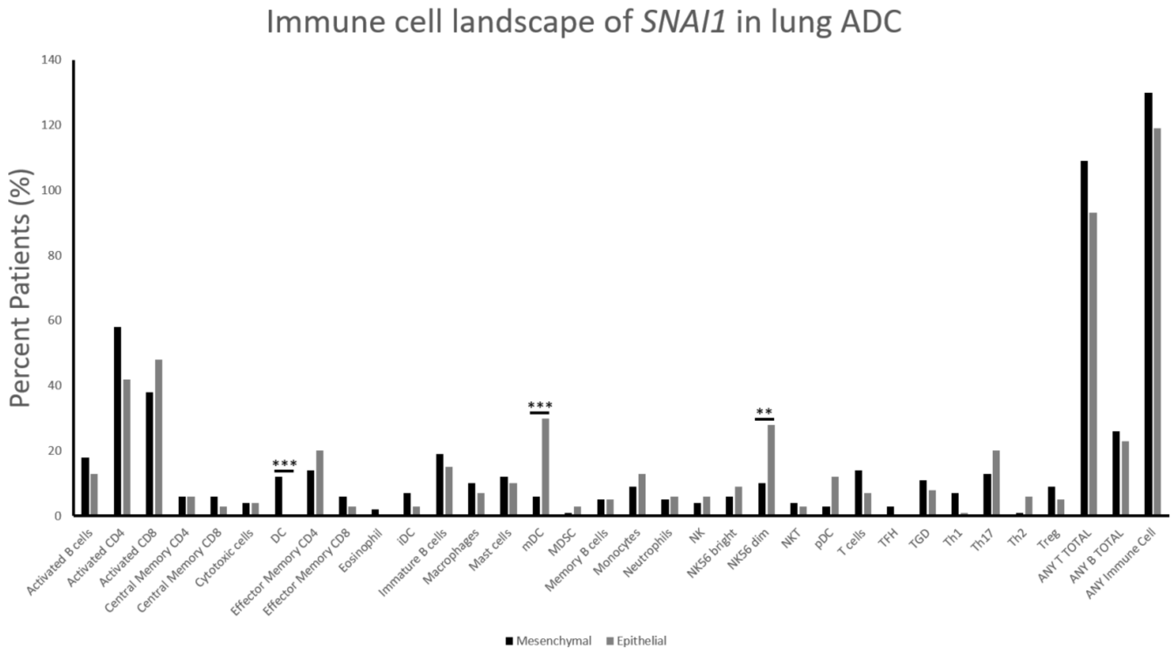

Supplementary Figure 10. D. Immune cell infiltration landscape of *SNAI1* in lung ADC by EMT score status. Immune infiltration of 31 distinct immune cells of 'mesenchymal' lung ADC compared to 'epithelial' lung ADC. * *p<0.05*, ** *p<0.01*, *** *p<0.001.*

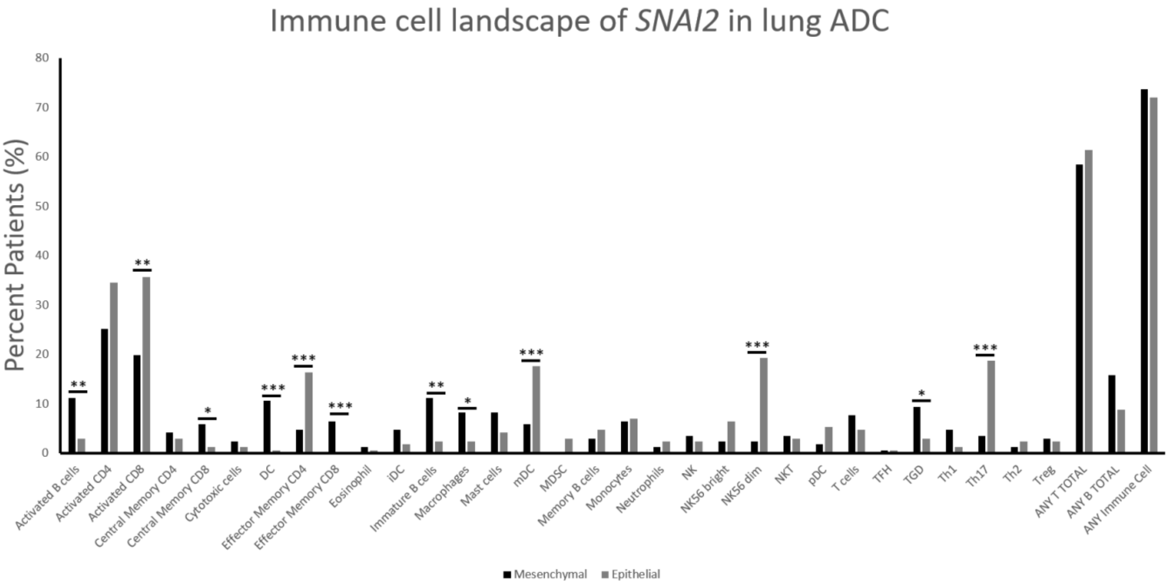

Supplementary Figure 10. E. Immune cell infiltration landscape of *SNAI2* in lung ADC by EMT score status. Immune infiltration of 31 distinct immune cells of ‘mesenchymal’ lung ADC compared to ‘epithelial’ lung ADC. * *p<0.05*, ** *p<0.01*, *** *p<0.001*.

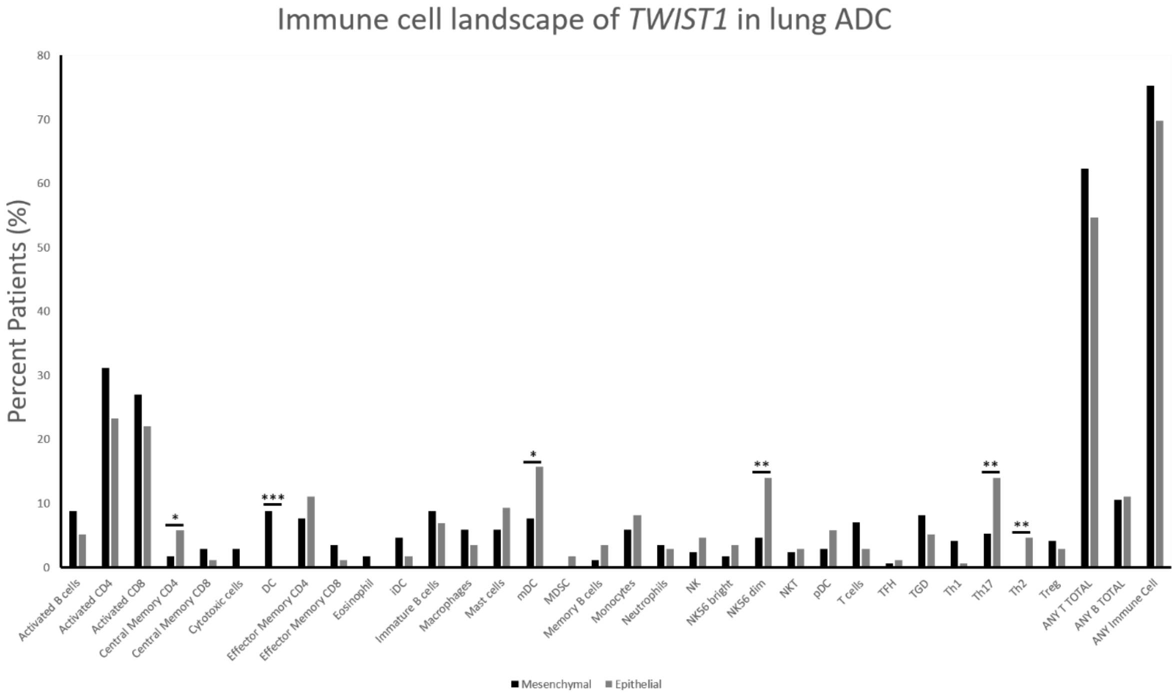

Supplementary Figure 10. F. Immune cell infiltration landscape of *TWIST1* in lung ADC by EMT score status. Immune infiltration of 31 distinct immune cells of 'mesenchymal' lung ADC compared to 'epithelial' lung ADC. * $p<0.05$, ** $p<0.01$, *** $p<0.001$.

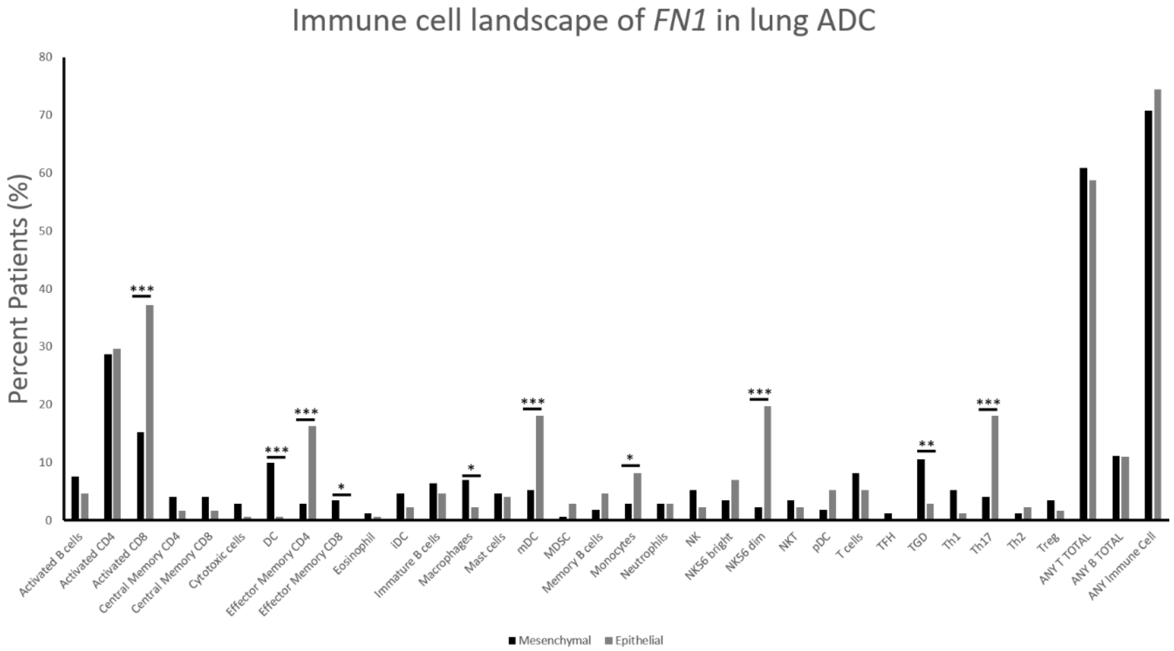

Supplementary Figure 10. G. Immune cell infiltration landscape of *FN1* in lung ADC by EMT score status. Immune infiltration of 31 distinct immune cells of 'mesenchymal' lung ADC compared to 'epithelial' lung ADC. * *p<0.05*, ** *p<0.01*, *** *p<0.001*.

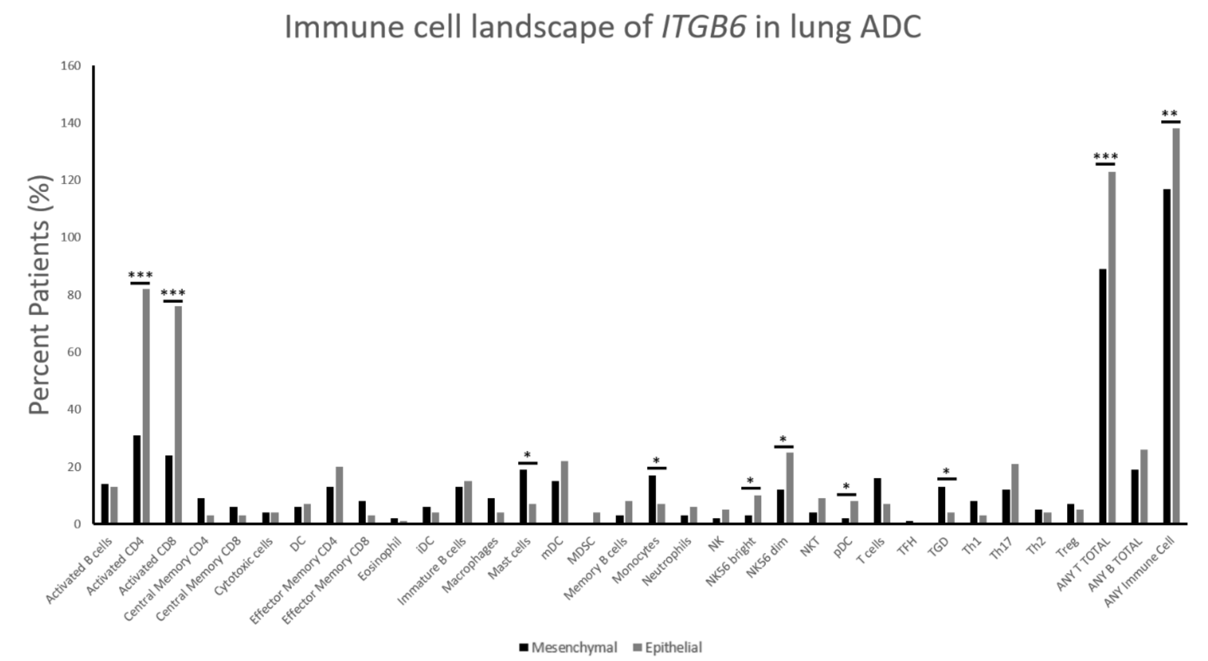

Supplementary Figure 10. H. Immune cell infiltration landscape of *ITGB6* in lung ADC by EMT score status. Immune infiltration of 31 distinct immune cells of 'mesenchymal' lung ADC compared to 'epithelial' lung ADC. * $p<0.05$, ** $p<0.01$, *** $p<0.001$.

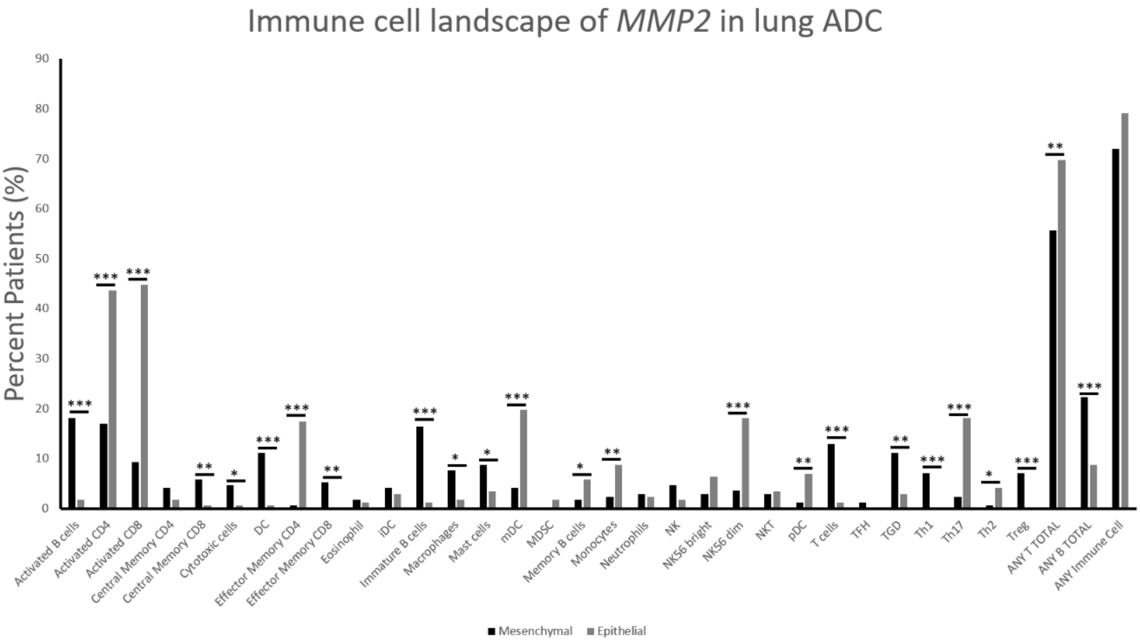

Supplementary Figure 10. I. Immune cell infiltration landscape of *MMP2* in lung ADC by EMT score status. Immune infiltration of 31 distinct immune cells of ‘mesenchymal’ lung ADC compared to ‘epithelial’ lung ADC. * *p<0.05*, ** *p<0.01*, *** *p<0.001*.

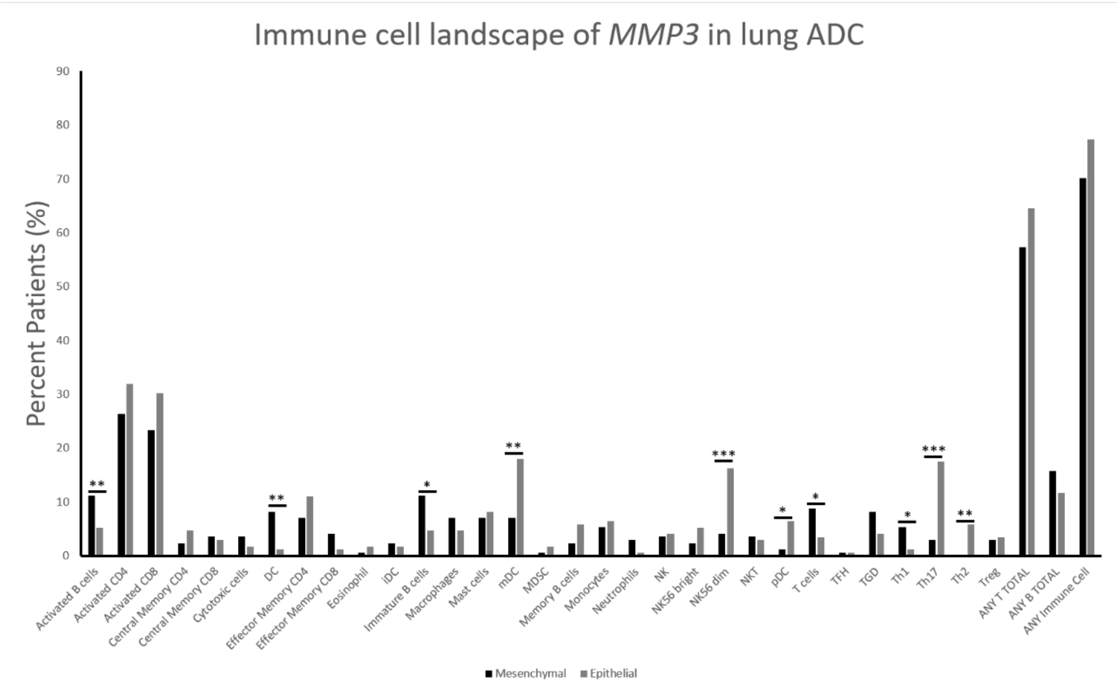

Supplementary Figure 10. J. Immune cell infiltration landscape of *MMP3* in lung ADC by EMT score status. Immune infiltration of 31 distinct immune cells of ‘mesenchymal’ lung ADC compared to ‘epithelial’ lung ADC. * *p<0.05*, ** *p<0.01*, *** *p<0.001*.

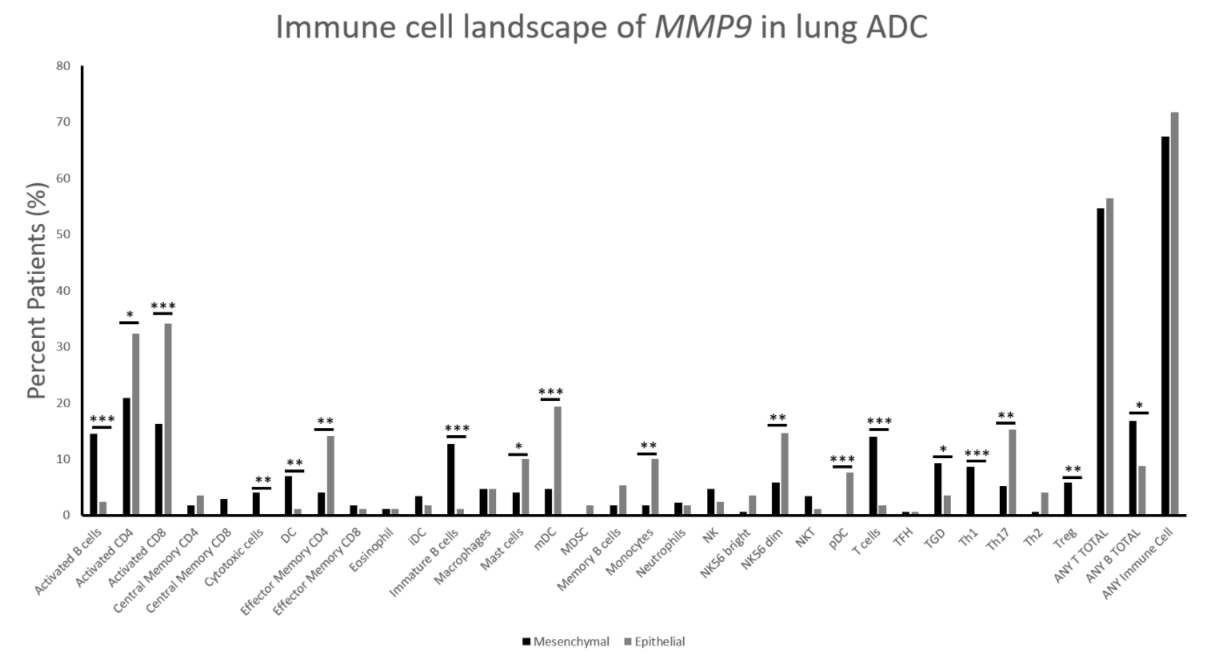

Supplementary Figure 10. K. Immune cell infiltration landscape of *MMP9* in lung ADC by EMT score status. Immune infiltration of 31 distinct immune cells of ‘mesenchymal’ lung ADC compared to ‘epithelial’ lung ADC. ** p<0.05, ** p<0.01, *** p<0.001.*

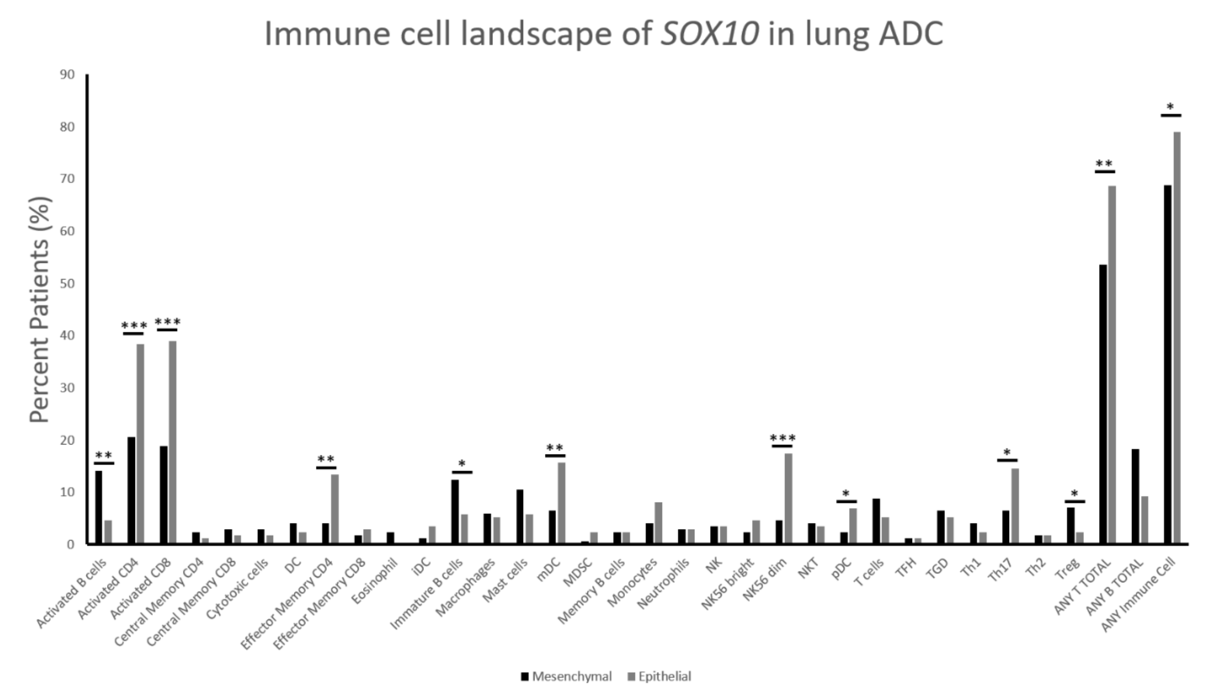

Supplementary Figure 10. L. Immune cell infiltration landscape of *SOX10* in lung ADC by EMT score status. Immune infiltration of 31 distinct immune cells of ‘mesenchymal’ lung ADC compared to ‘epithelial’ lung ADC. * $p<0.05$, ** $p<0.01$, *** $p<0.001$.

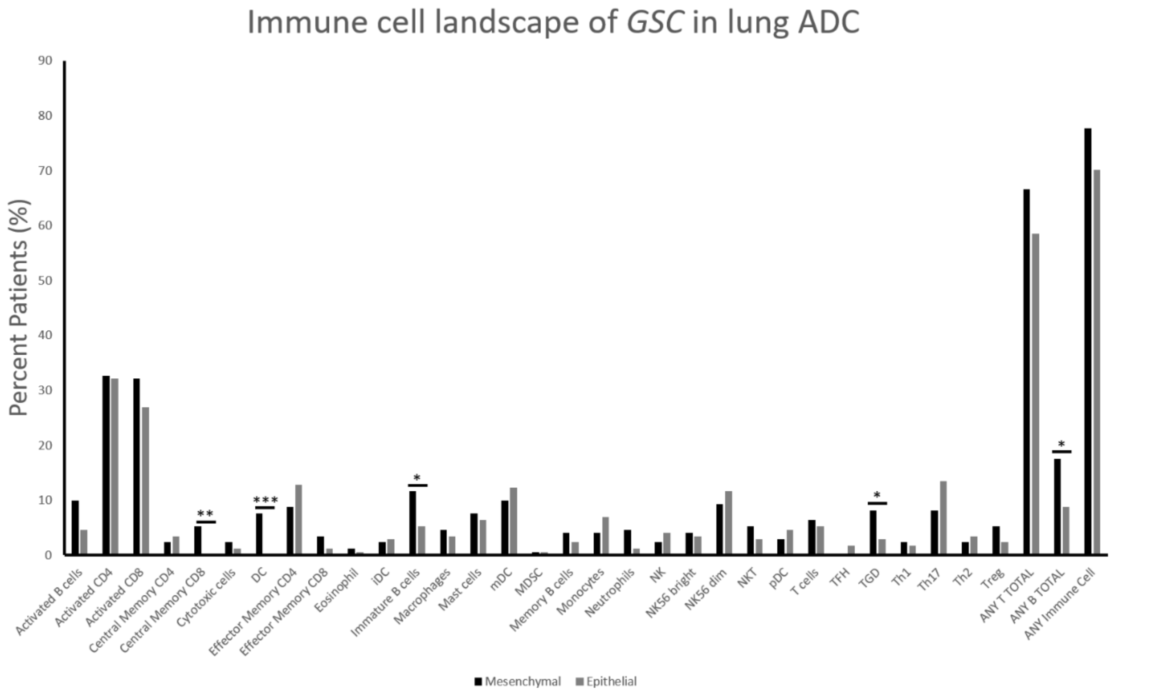

Supplementary Figure 10. M. Immune cell infiltration landscape of *GSC* in lung ADC by EMT score status. Immune infiltration of 31 distinct immune cells of 'mesenchymal' lung ADC compared to 'epithelial' lung ADC. * *p<0.05*, ** *p<0.01*, *** *p<0.001*.

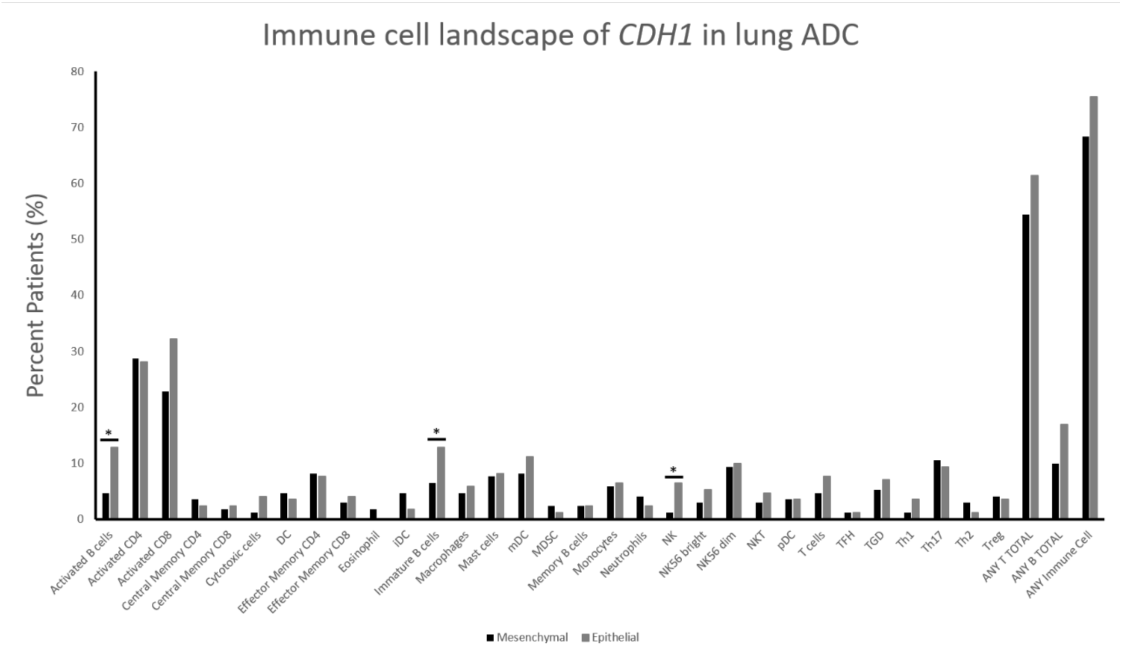

Supplementary Figure 10. N. Immune cell infiltration landscape of *CDH1* in lung ADC by EMT score status. Immune infiltration of 31 distinct immune cells of ‘mesenchymal’ lung ADC compared to ‘epithelial’ lung ADC. * *p<0.05*, ** *p<0.01*, *** *p<0.001*.

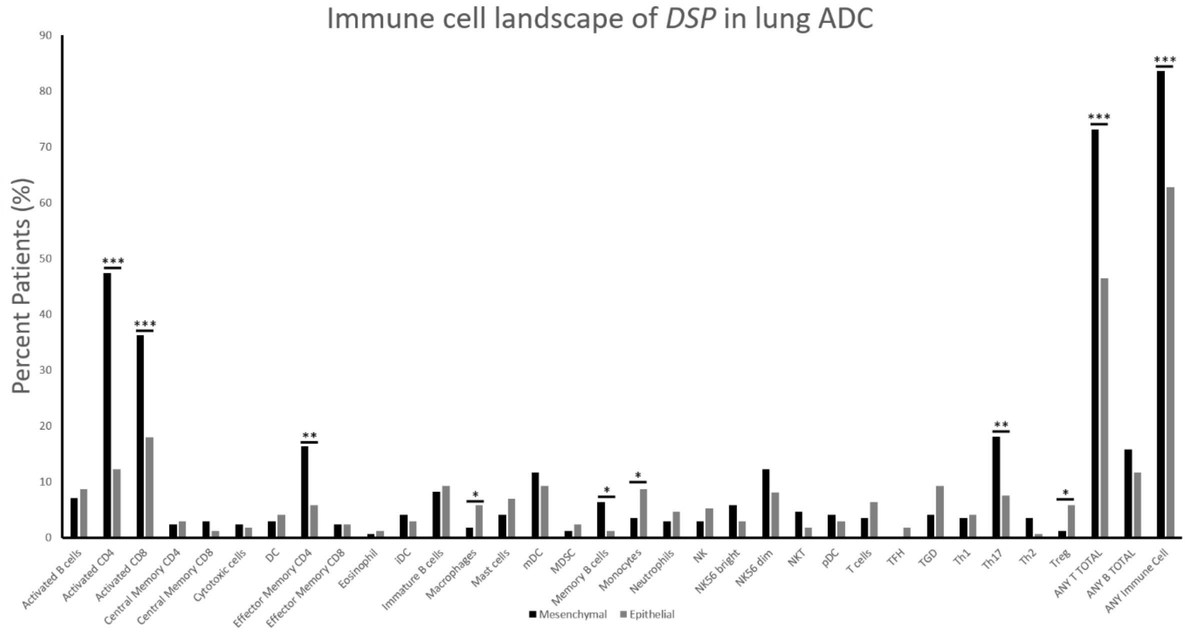

Supplementary Figure 10. O. Immune cell infiltration landscape of *DSP* in lung ADC by EMT score status. Immune infiltration of 31 distinct immune cells of 'mesenchymal' lung ADC compared to 'epithelial' lung ADC. * *p<0.05*, ** *p<0.01*, *** *p<0.001*.

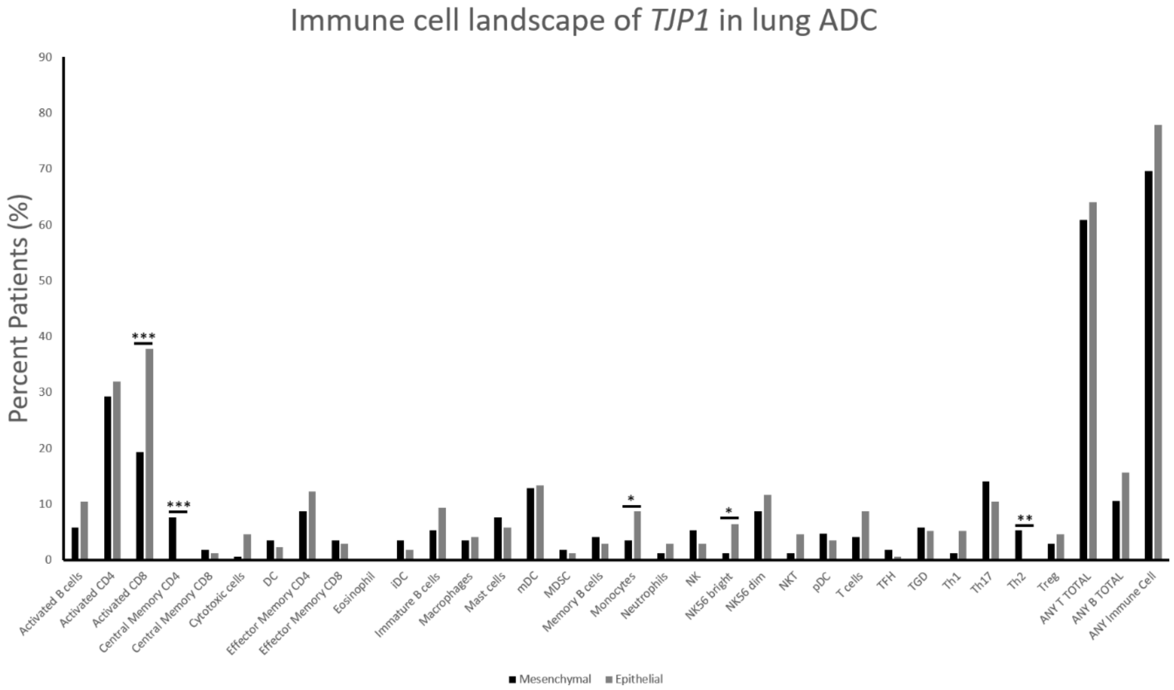

Supplementary Figure 10. P. Immune cell infiltration landscape of *DSP* in lung ADC by EMT score status. Immune infiltration of 31 distinct immune cells of 'mesenchymal' lung ADC compared to 'epithelial' lung ADC. * *p<0.05*, ** *p<0.01*, *** *p<0.001*.

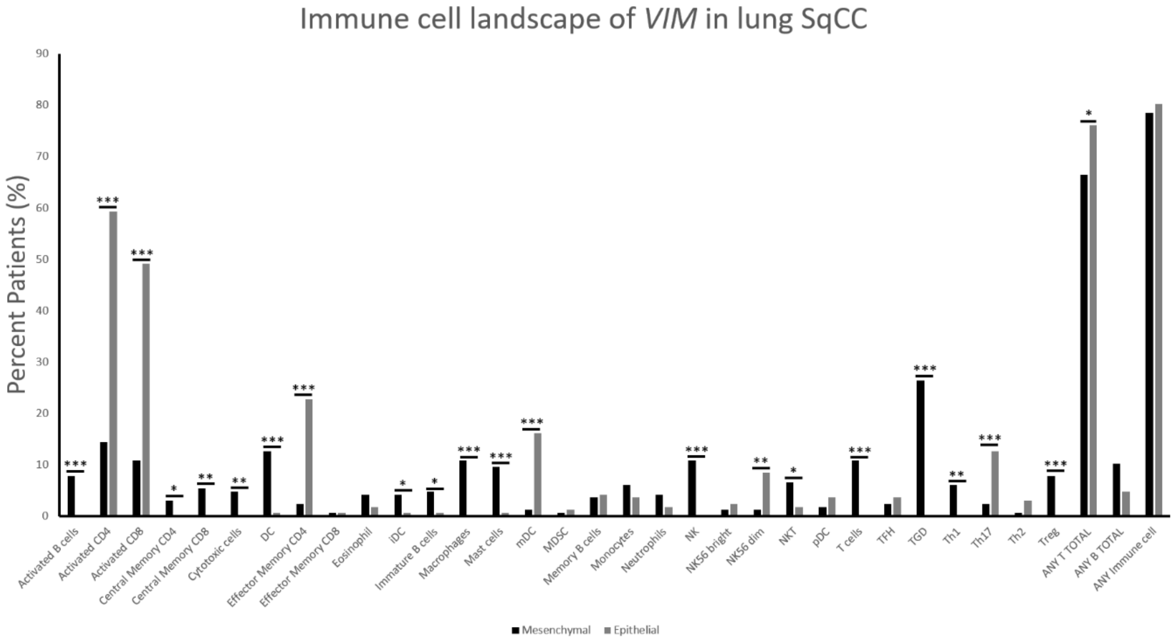

Supplementary Figure 11. A. Immune cell infiltration landscape of *VIM* in lung SqCC by EMT score status. Immune infiltration of 31 distinct immune cells of ‘mesenchymal’ lung SqCC compared to ‘epithelial’ lung SqCC. * $p<0.05$, ** $p<0.01$, *** $p<0.001$.

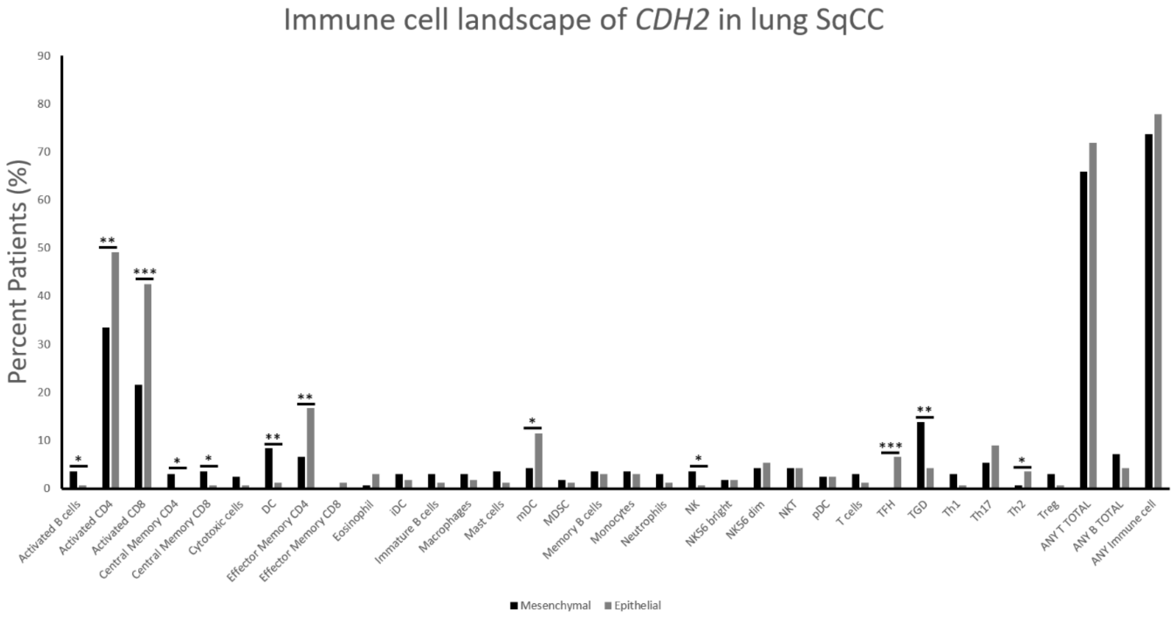

Supplementary Figure 11. B. Immune cell infiltration landscape of *CDH2* in lung SqCC by EMT score status. Immune infiltration of 31 distinct immune cells of ‘mesenchymal’ lung SqCC compared to ‘epithelial’ lung SqCC. * *$p<0.05$, ** $p<0.01$, *** $p<0.001$*.

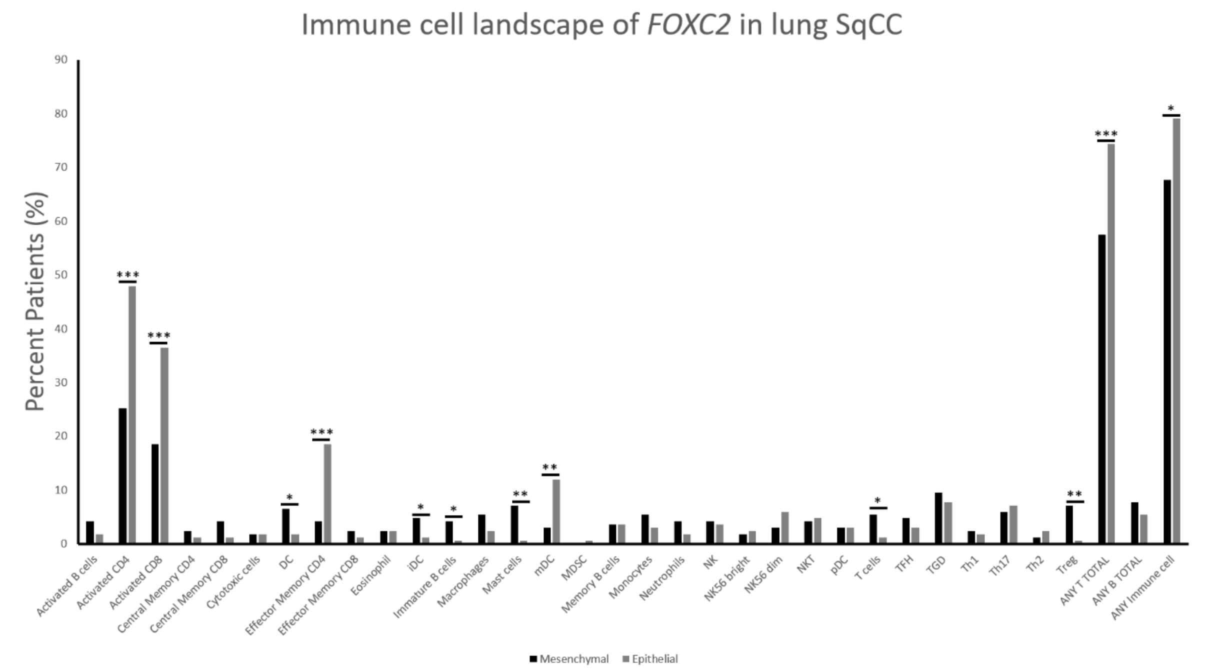

Supplementary Figure 11. C. Immune cell infiltration landscape of *FOXC2* in lung SqCC by EMT score status. Immune infiltration of 31 distinct immune cells of ‘mesenchymal’ lung SqCC compared to ‘epithelial’ lung SqCC. * *p<0.05*, ** *p<0.01*, *** *p<0.001.*

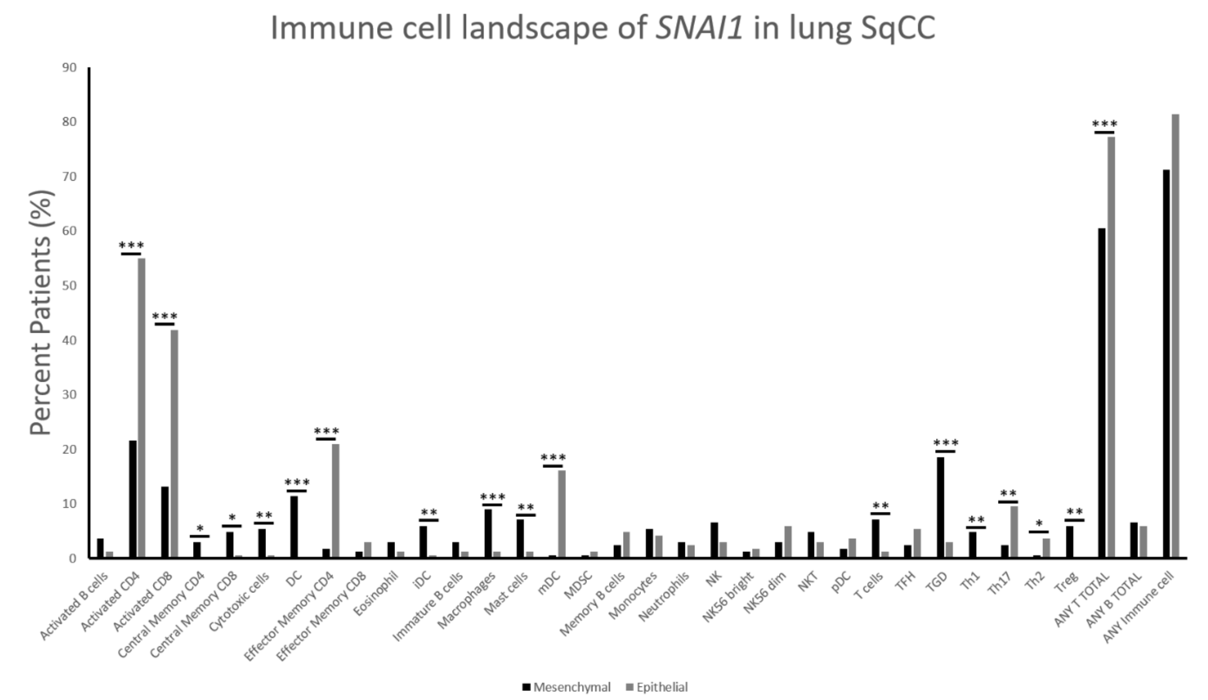

Supplementary Figure 11. D. Immune cell infiltration landscape of *SNAI1* in lung SqCC by EMT score status. Immune infiltration of 31 distinct immune cells of 'mesenchymal' lung SqCC compared to 'epithelial' lung SqCC. * *p<0.05*, ** *p<0.01*, *** *p<0.001*.

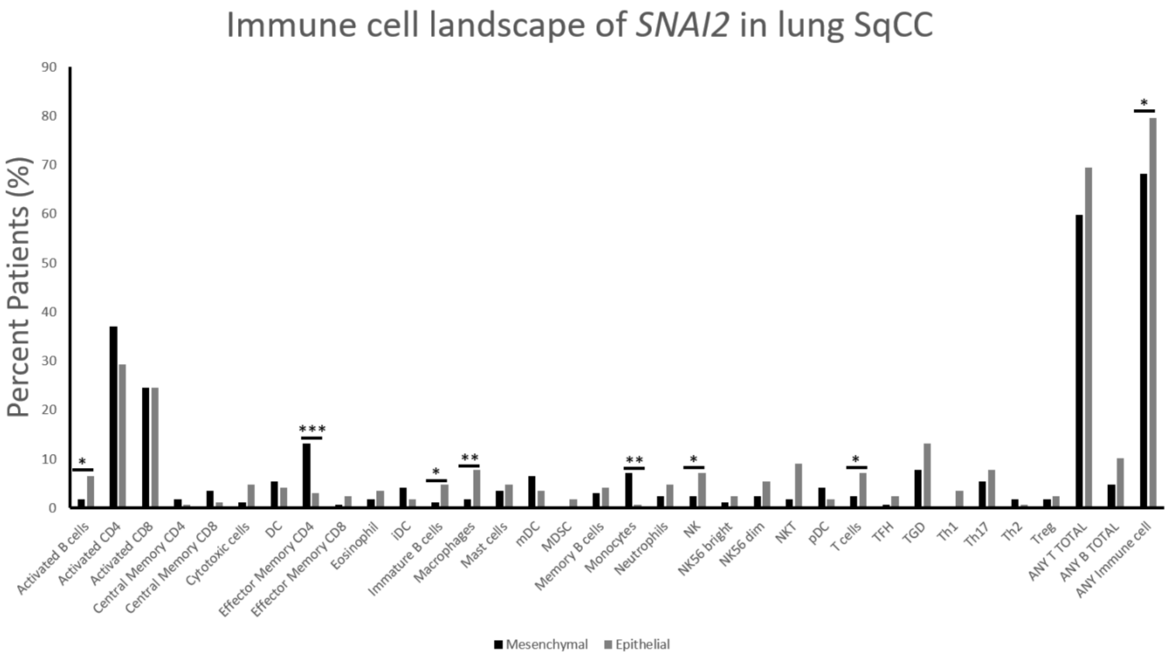

Supplementary Figure 11. E. Immune cell infiltration landscape of *SNAI2* in lung SqCC by EMT score status. Immune infiltration of 31 distinct immune cells of 'mesenchymal' lung SqCC compared to 'epithelial' lung SqCC. * *p<0.05, *** *p<0.01*, *** *p<0.001*.

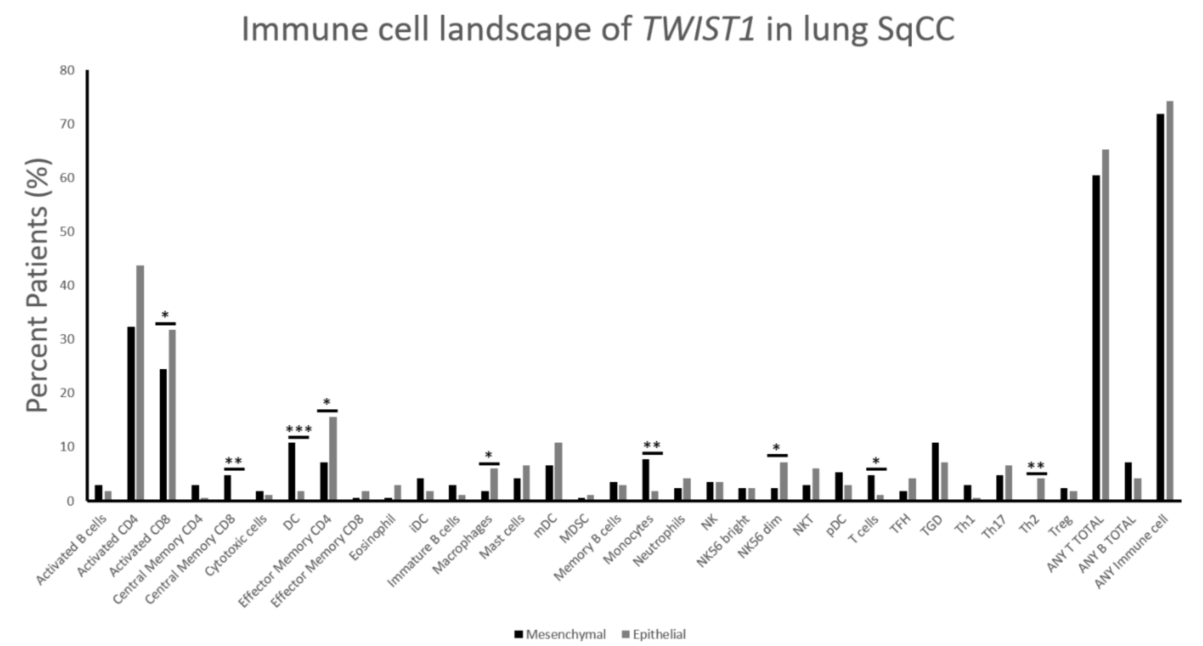

Supplementary Figure 11. F. Immune cell infiltration landscape of *TWIST1* in lung SqCC by EMT score status. Immune infiltration of 31 distinct immune cells of 'mesenchymal' lung SqCC compared to 'epithelial' lung SqCC. * *p<0.05,* ** *p<0.01,* *** *p<0.001.*

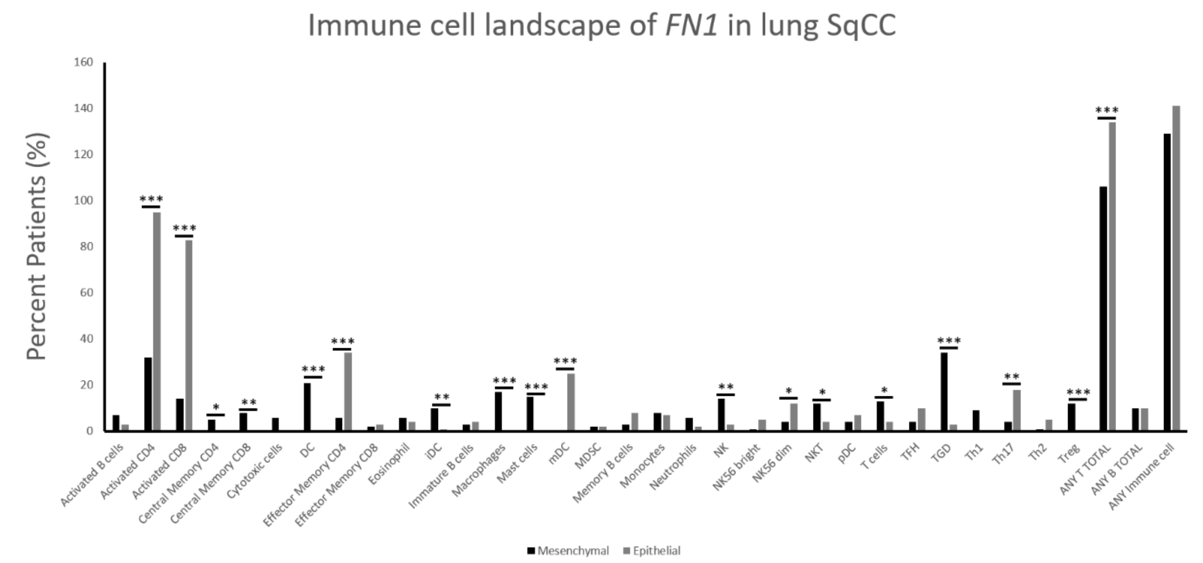

Supplementary Figure 11. G. Immune cell infiltration landscape of *FN1* in lung SqCC by EMT score status. Immune infiltration of 31 distinct immune cells of 'mesenchymal' lung SqCC compared to 'epithelial' lung SqCC. * *p<0.05*, ** *p<0.01*, *** *p<0.001*.

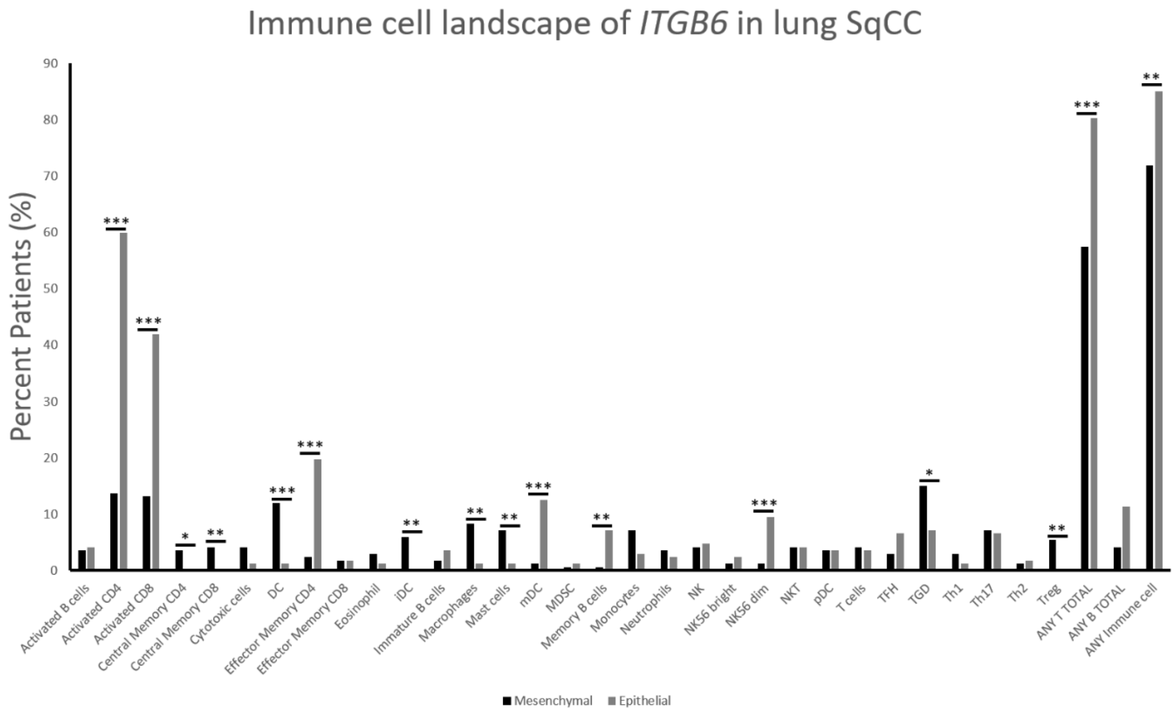

Supplementary Figure 11. H. Immune cell infiltration landscape of *ITGB6* in lung SqCC by EMT score status. Immune infiltration of 31 distinct immune cells of 'mesenchymal' lung SqCC compared to 'epithelial' lung SqCC. * *p<0.05, ** p<0.01, *** p<0.001.*

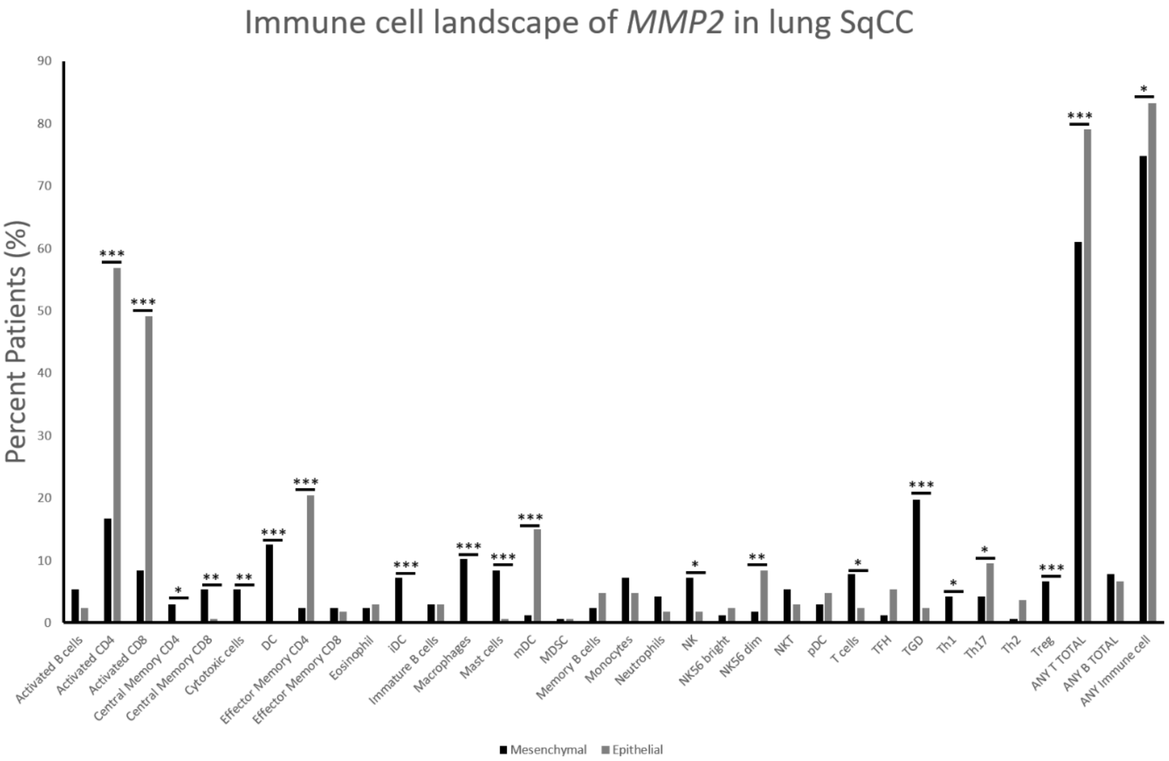

Supplementary Figure 11. I. Immune cell infiltration landscape of *MMP2* in lung SqCC by EMT score status. Immune infiltration of 31 distinct immune cells of ‘mesenchymal’ lung SqCC compared to ‘epithelial’ lung SqCC. * $p<0.05$, ** $p<0.01$, *** $p<0.001$.

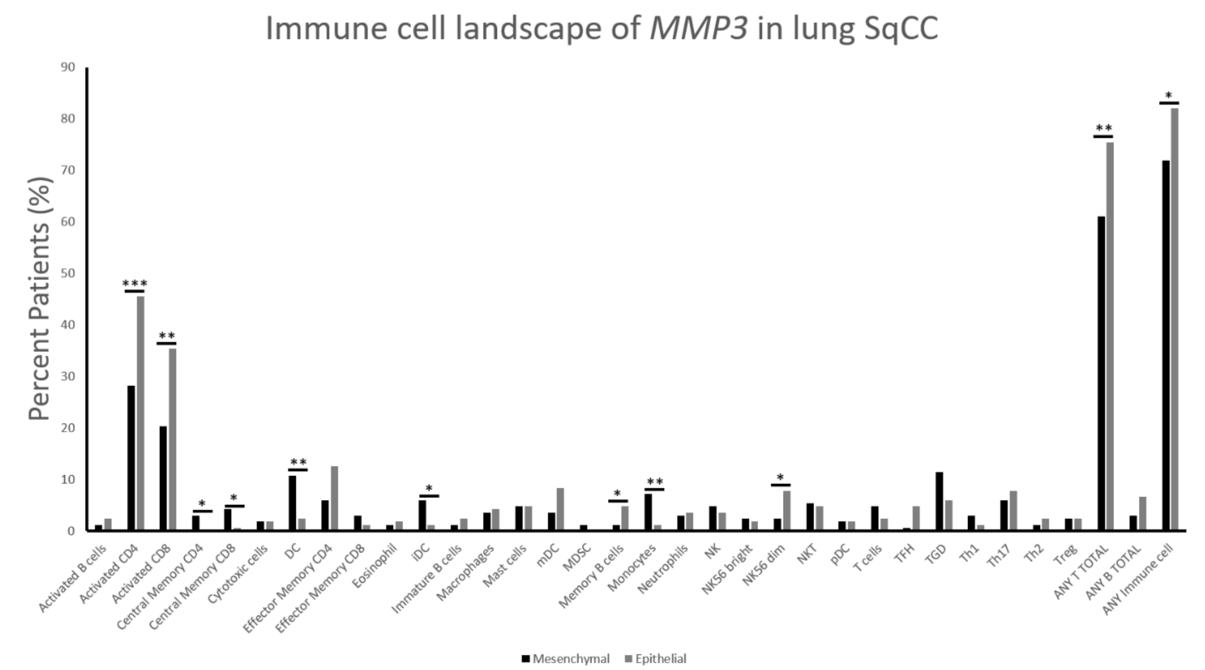

Supplementary Figure 11. J. Immune cell infiltration landscape of *MMP3* in lung SqCC by EMT score status. Immune infiltration of 31 distinct immune cells of ‘mesenchymal’ lung SqCC compared to ‘epithelial’ lung SqCC. * *p<0.05*, ** *p<0.01*, *** *p<0.001*.

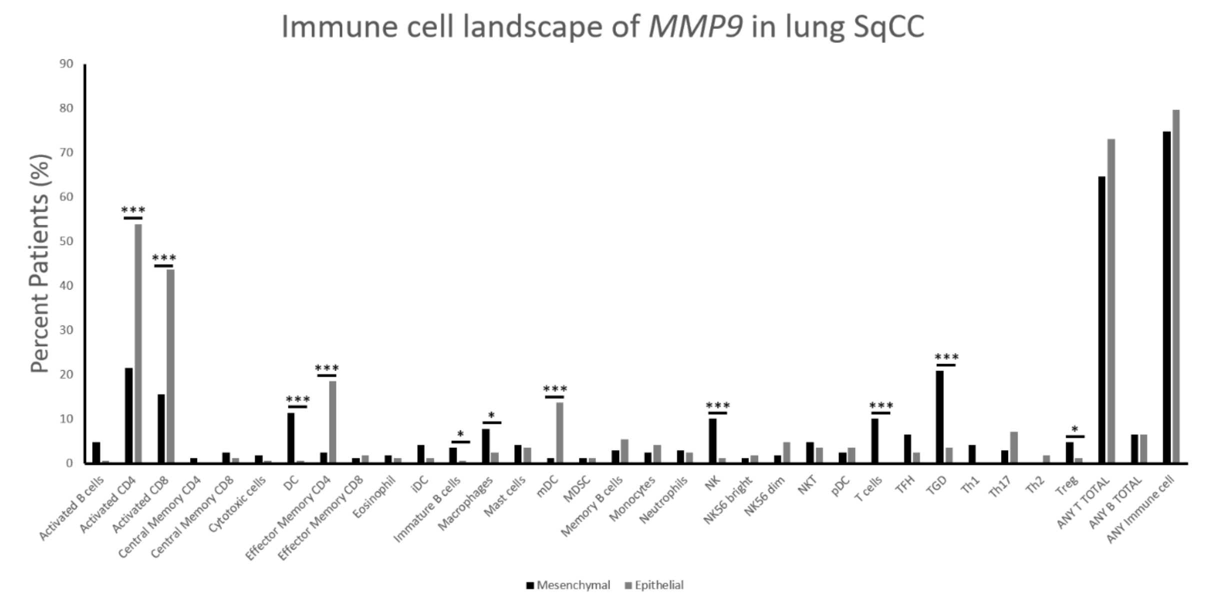

Supplementary Figure 11. K. Immune cell infiltration landscape of *MMP9* in lung SqCC by EMT score status. Immune infiltration of 31 distinct immune cells of 'mesenchymal' lung SqCC compared to 'epithelial' lung SqCC. ** p<0.05, ** p<0.01, *** p<0.001.*

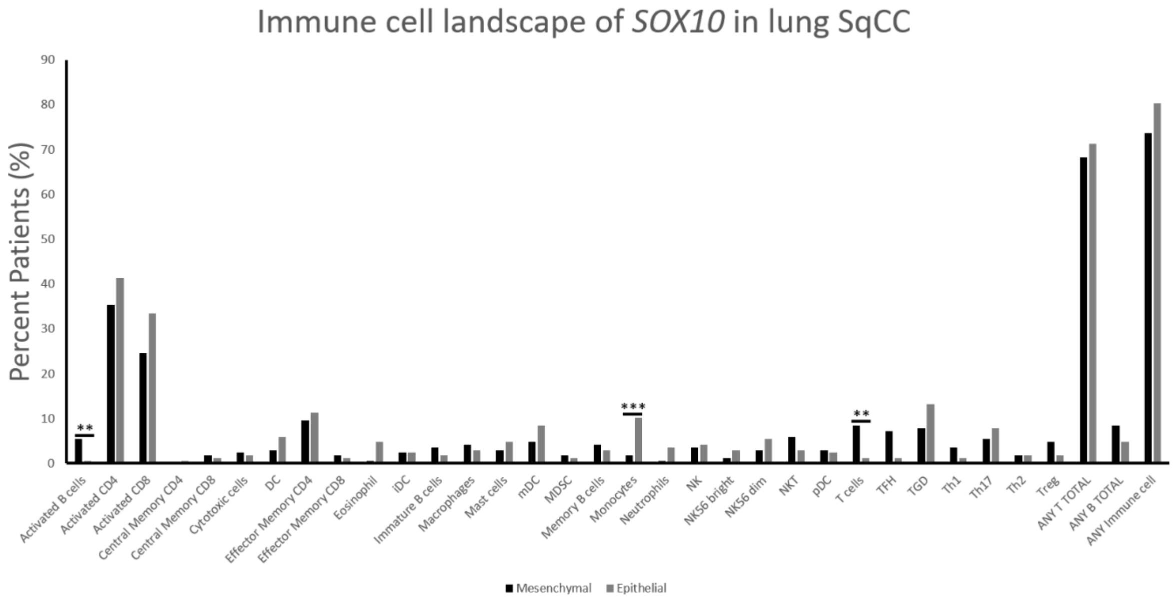

Supplementary Figure 11. L. Immune cell infiltration landscape of *SOX10* in lung SqCC by EMT score status. Immune infiltration of 31 distinct immune cells of 'mesenchymal' lung SqCC compared to 'epithelial' lung SqCC. * *p<0.05, *** *p<0.01, **** *p<0.001*.

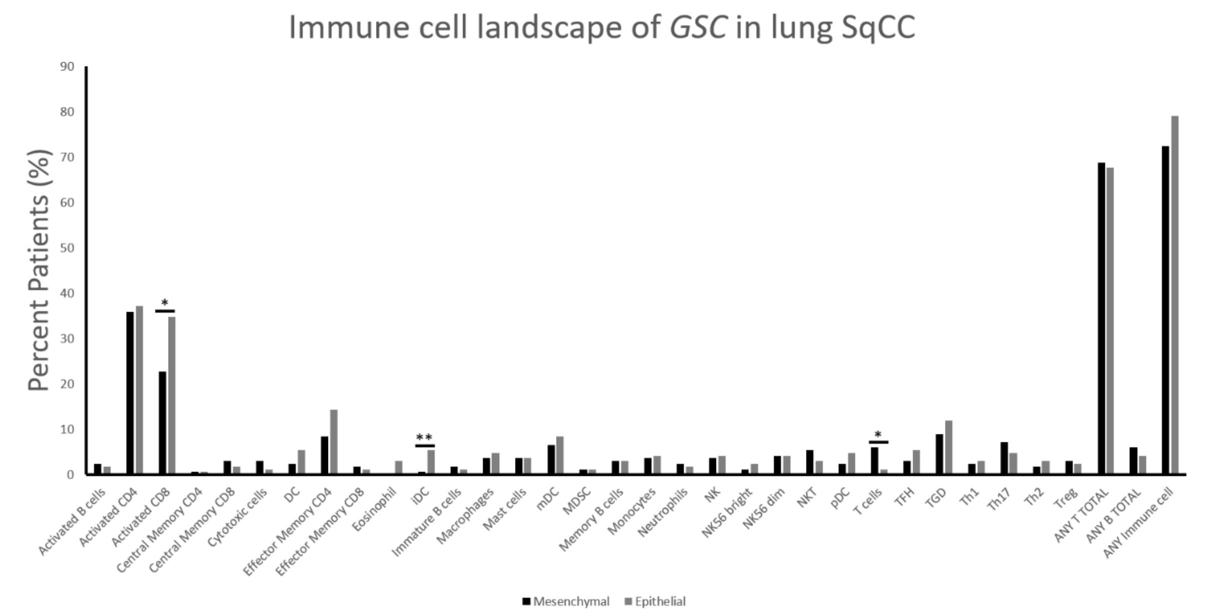

Supplementary Figure 11. M. Immune cell infiltration landscape of *GSC* in lung SqCC by EMT score status. Immune infiltration of 31 distinct immune cells of 'mesenchymal' lung SqCC compared to 'epithelial' lung SqCC. * *p<0.05*, ** *p<0.01*, *** *p<0.001*.

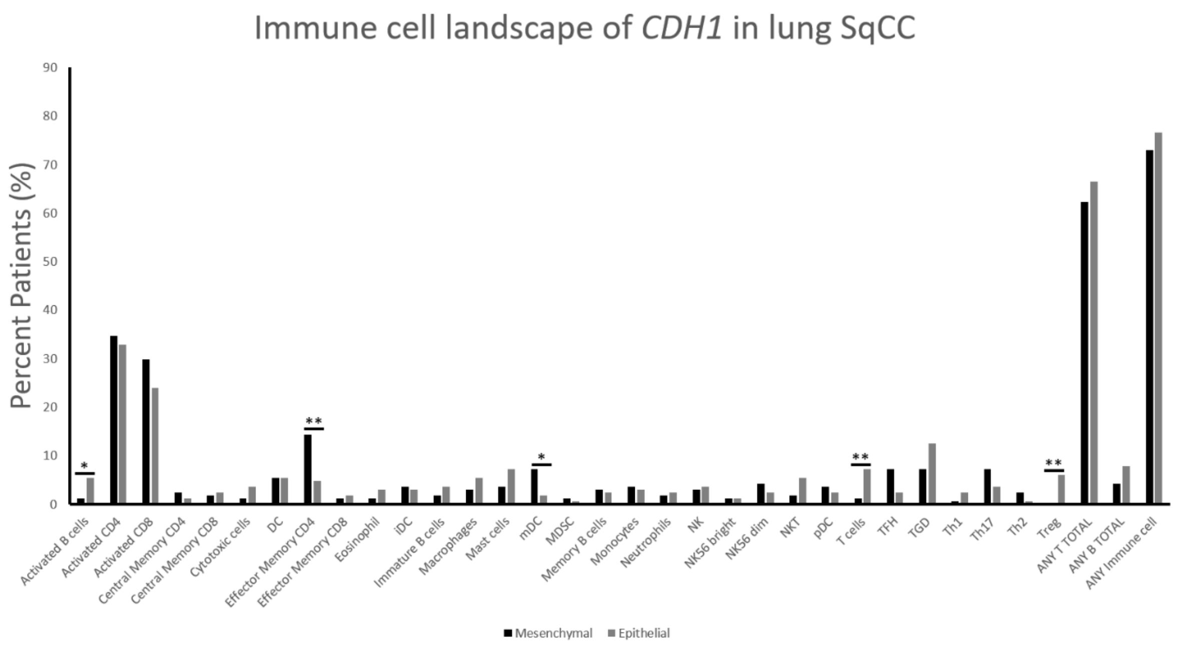

Supplementary Figure 11. N. Immune cell infiltration landscape of *CDH1* in lung SqCC by EMT score status. Immune infiltration of 31 distinct immune cells of 'mesenchymal' lung SqCC compared to 'epithelial' lung SqCC. * $p<0.05$, ** $p<0.01$, *** $p<0.001$.

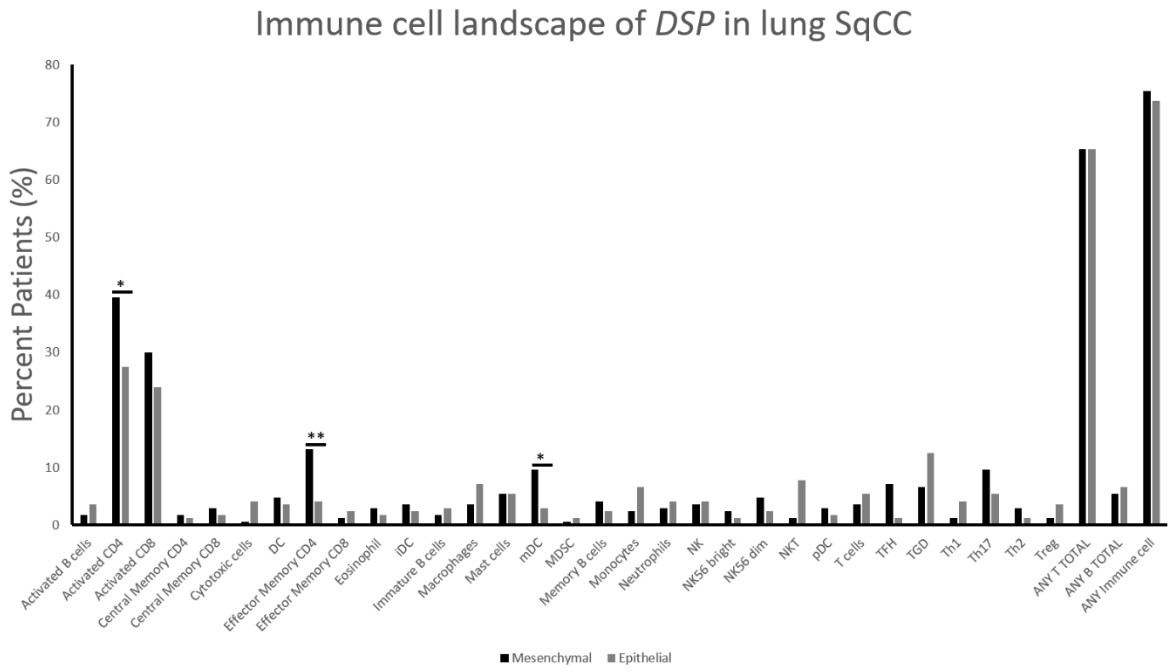

Supplementary Figure 11. O. Immune cell infiltration landscape of *DSP* in lung SqCC by EMT score status. Immune infiltration of 31 distinct immune cells of ‘mesenchymal’ lung SqCC compared to ‘epithelial’ lung SqCC. * $p<0.05$, ** $p<0.01$, *** $p<0.001$.

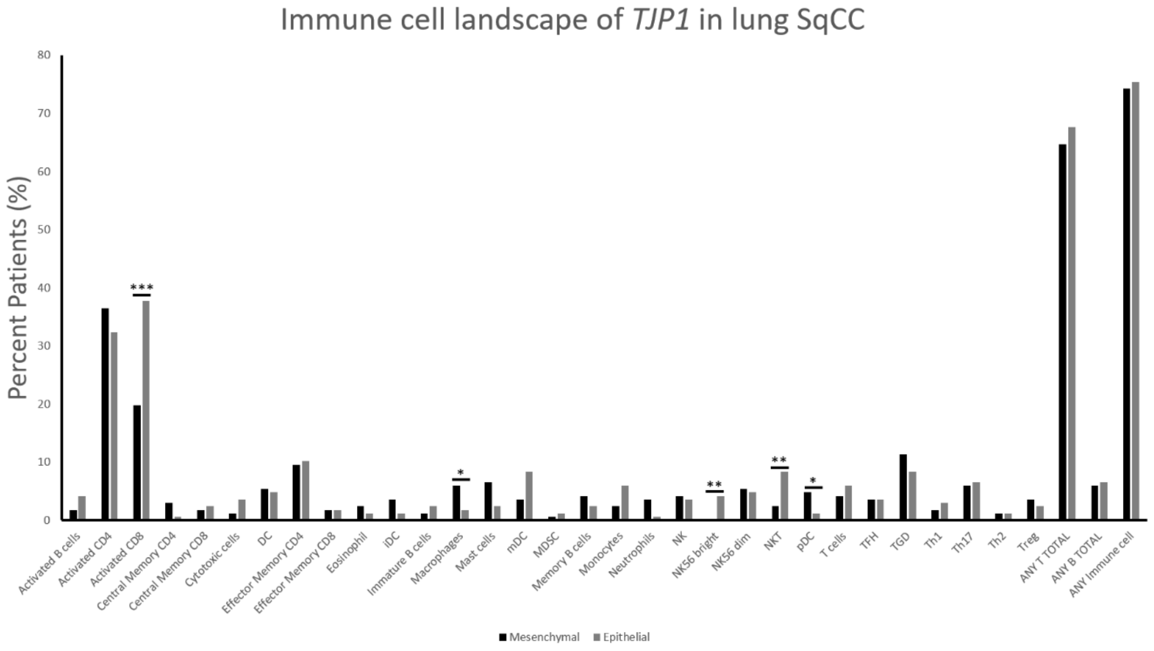

Supplementary Figure 11. P. Immune cell infiltration landscape of *TJP1* in lung SqCC by EMT score status. Immune infiltration of 31 distinct immune cells of 'mesenchymal' lung SqCC compared to 'epithelial' lung SqCC. * *p<0.05*, ** *p<0.01*, *** *p<0.001*.

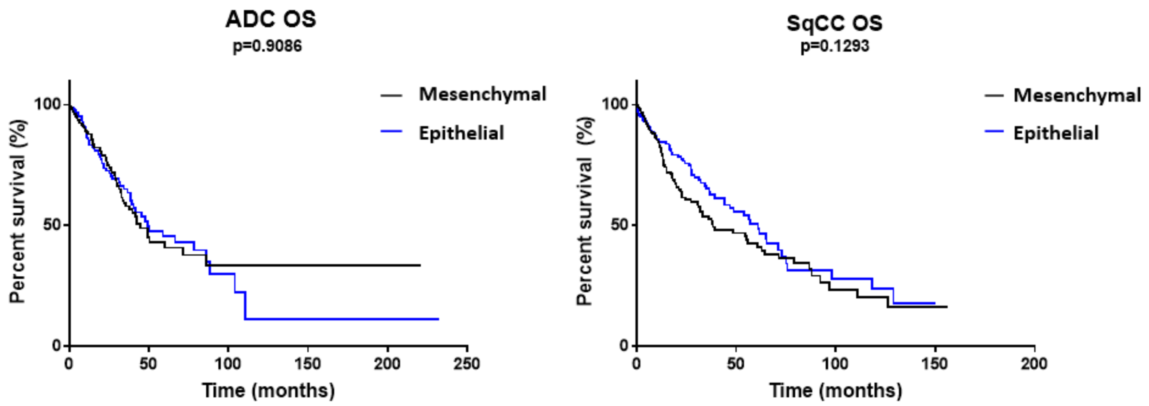

Supplementary Figure 12. A. Overall survival of mesenchymal and epithelial lung ADC, SqCC.

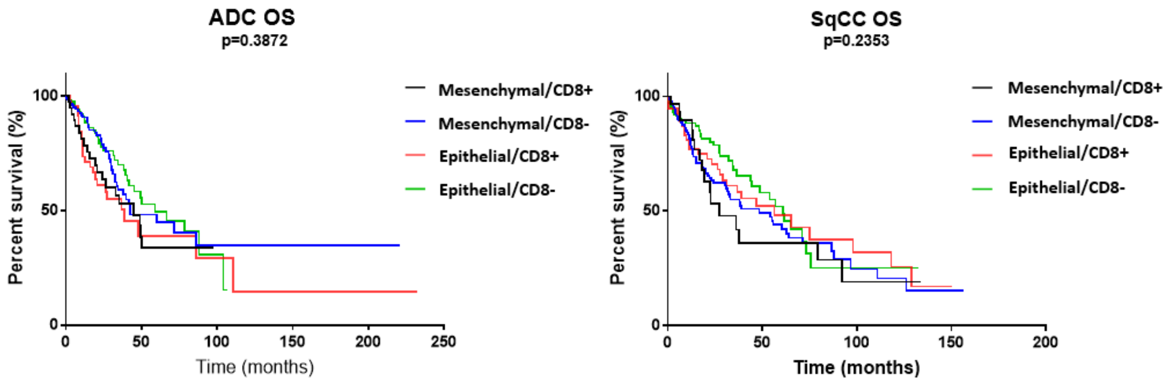

Supplementary Figure 12. B. Overall survival of mesenchymal and epithelial lung ADC, SqCC according to infiltration of CD8 T-cells.
